# Effectiveness of S-1–Based Chemoradiotherapy in Patients 70 Years and Older With Esophageal Squamous Cell Carcinoma: A Randomized Clinical Trial

**DOI:** 10.1001/jamanetworkopen.2023.12625

**Published:** 2023-05-17

**Authors:** Xin Wang, Weiming Han, Wencheng Zhang, Xiaomin Wang, Xiaolin Ge, Yu Lin, Haiwen Zhou, Miaomiao Hu, Wei Wang, Ke Liu, Jianchao Lu, Shuai Qie, Jihong Zhang, Wei Deng, Lan Wang, Chun Han, Minghe Li, Kaixian Zhang, Ling Li, Qifeng Wang, Hongyun Shi, Zhilong Yu, Yidian Zhao, Xinchen Sun, Yonggang Shi, Qingsong Pang, Zongmei Zhou, Jun Liang, Dongfu Chen, Qinfu Feng, Nan Bi, Tao Zhang, Lei Deng, Wenqing Wang, Wenyang Liu, Jianyang Wang, Yirui Zhai, Junjie Wang, Wanqing Chen, Junqiang Chen, Zefen Xiao

**Affiliations:** 1 Department of Radiation Oncology, National Cancer Center, National Clinical Research Center for Cancer, Cancer Hospital, Chinese Academy of Medical Sciences and Peking Union Medical College, Beijing, China; 2 Department of Radiation Oncology, Tianjin Medical University Cancer Institute and Hospital, National Clinical Research Center for Cancer, Tianjin, China; 3 Department of Radiation Oncology, Anyang Cancer Hospital, Anyang, China; 4 Department of Radiation Oncology, Jiangsu Province Hospital, the First Affiliated Hospital with Nanjing Medical University, Nanjing, China; 5 Department of Radiation Oncology, Clinical Oncology School of Fujian Medical University, Fujian Cancer Hospital, Fuzhou, China; 6 Department of Oncology, Tengzhou Central People’s Hospital, Tengzhou, China; 7 Department of Radiation Oncology, the First Affiliated Hospital of Zhengzhou University, Zhengzhou, China; 8 Department of Radiation Oncology, Sichuan Cancer Hospital and Institution, Sichuan Cancer Center, School of Medicine, University of Electronic Science and Technology of China, Radiation Oncology Key Laboratory of Sichuan Province, Chengdu, China; 9 Department of Radiation Oncology, Affiliated Hospital of Hebei University, Baoding, China; 10 Department of Radiation Oncology, The Affiliated Hospital of Inner Mongolia Medical University, Hohhot, China; 11 Department of Radiation Oncology, Key Laboratory of Carcinogenesis and Translational Research (Ministry of Education/Beijing), Peking University Cancer Hospital and Institute, Beijing, China; 12 Department of Radiation Oncology, the Fourth Hospital of Hebei Medical University, Shijiazhuang, China; 13 Department of Radiation Oncology, Peking University Third Hospital, Beijing, China; 14 Office of Cancer Screening, National Cancer Center, National Clinical Research Center for Cancer, Cancer Hospital, Chinese Academy of Medical Sciences and Peking Union Medical College, Beijing, China

## Abstract

**Importance:**

Double-agent intravenous chemotherapy concurrent with radiotherapy is the standard of care for patients with inoperable esophageal cancer. However, patients tend to tolerate intravenous chemotherapy less well with age and comorbidities. It is essential to find a better treatment modality that improves survival outcomes without reducing the quality of life.

**Objective:**

To evaluate the effectiveness of simultaneous integrated boost radiotherapy (SIB-RT) with concurrent and consolidated oral S-1 chemotherapy for patients aged 70 years and older with inoperable esophageal squamous cell carcinoma (ESCC).

**Design, Setting, and Participants:**

This multicenter, phase III randomized clinical trial was conducted between March 2017 and April 2020 in 10 centers in China. Patients with inoperable, locally advanced, clinical stage II to IV ESCC were enrolled and randomized to receive SIB-RT concurrent with and followed by oral S-1 chemotherapy (CRTCT group) or SIB-RT alone (RT group). Data analysis was completed on March 22, 2022.

**Interventions:**

In both groups, the planning gross tumor volume was administered with radiation dose of 59.92 Gy and the planning target volume was administered with radiation dose of 50.4 Gy, in 28 fractions each. In the CRTCT group, concurrent S-1 was administered on radiotherapy days, and consolidated S-1 was administered at 4 to 8 weeks after SIB-RT.

**Main Outcomes and Measures:**

The primary end point was overall survival (OS) of the intent-to-treat population. Secondary end points were progression-free survival (PFS) and toxicity profile.

**Results:**

A total of 330 patients (median [IQR] age, 75.5 [72-79] years; 220 [66.7%] male patients) were included, with 146 patients randomized to the RT group and 184 randomized to the CRTCT group. A total of 107 patients (73.3%) in the RT group and 121 patients (67.9%) in the CRTCT group were clinically diagnosed with stage III to IV disease. At the time of analysis of the 330 patients in the intent-to treat-population (March 22, 2022), OS was improved in the CRTCT group compared with the RT group at 1 year (72.2% vs 62.3%) and 3 years (46.2% vs 33.9%; log-rank *P* = .02). PFS was similarly improved in the CRTCT group compared with the RT group at 1 year (60.8% vs 49.3%) and 3 years (37.3% vs 27.9%; log-rank *P* = .04). There was no significant difference in the incidence of treatment-related toxic effects higher than grade 3 between the 2 groups. Grade 5 toxic effects occurred in each group, including 1 patient who experienced myelosuppression and 4 patients with pneumonitis in the RT group and 3 patients with pneumonitis and 2 patients with fever in the CRTCT group.

**Conclusions and Relevance:**

These findings suggest that oral S-1 chemotherapy administered with SIB-RT should be considered as an alternative treatment option for patients aged 70 years and older with inoperable ESCC, since it improved survival outcomes without additional treatment-related toxic effects compared with SIB-RT alone.

**Trial Registration:**

ClinicalTrials.gov Identifier: NCT02979691

## Key Points

**Question:**

Is oral S-1 chemotherapy with simultaneous integrated boost radiotherapy (SIB-RT) effective and safe for patients aged 70 years and older with inoperable esophageal cancer (EC)?

**Findings:**

In this randomized clinical trial of 330 patients aged 70 years and older with EC who were randomly assigned to receive SIB-RT with concurrent and consolidated oral S-1 chemotherapy (CRTCT) or SIB-RT alone, 3-year overall survival and progression-free survival of participants in the CRTCT group were superior to that of SIB-RT group. The incidence of grade 3 to 5 toxic effects was similar in both groups.

**Meaning:**

These findings suggest that oral S-1 chemotherapy with SIB-RT should be considered as an alternative treatment option for patients aged 70 years and older with inoperable EC to improve survival outcomes with acceptable treatment-related toxic effects.

## Teaser

This randomized clinical trial compares the efficacy and safety of simultaneous integrated boost radiotherapy with concurrent and consolidated oral S-1 chemotherapy vs radiotherapy alone for patients aged 70 years and older with inoperable esophageal squamous cell carcinoma.

## Introduction

The incidence of esophageal squamous cell carcinoma (ESCC) has increased steadily among patients aged 70 years and older, who account for 30% to 40% of all patients with ESCC worldwide.^1^ Based on the results from the Radiation Therapy Oncology Group (RTOG) trial 85-01,^2^ concurrent chemoradiotherapy has become the standard treatment option for inoperable locally advanced esophageal carcinoma. Since patients tend to tolerate intravenous chemotherapy less well with age and comorbidities,^3,4^ the standard radiotherapy (RT) with concurrent double-agent intravenous chemotherapy can be difficult for patients aged 70 years and older.

In addition to the advanced staging technique leading to better patient selection, advanced radiation techniques, extensive application of intensity-modulated RT (IMRT), and image-guided RT have improved survival outcomes compared with the era of conformal RT.^5,6^ For patients receiving RT alone, the median survival time is approximately 20.7 months with the use of IMRT. In several previous studies, patients received irradiation to the planning target volume (PTV), with a subsequent boost in the planning gross tumor volume (PGTV).^5,7,8^ In contrast, simultaneous integrated boost RT (SIB-RT) could simultaneously deliver irradiation to both PTV and PGTV; avoid resimulation, recontouring, and replanning; and shorten the overall treatment course. Based on our previous phase I/II trial,^9,10^ SIB-RT can be delivered with a radiation dose of 50.40 Gy in 28 fractions (equivalent dose in 2 Gy [EQD2], 49.56 Gy) delivered to the involved lymphatic drainage region and a simultaneous radiation dose of 59.92 Gy in 28 fractions (EQD2, 60.62 Gy) delivered to the primary tumor and metastatic regional lymph nodes and may be an alternative treatment option over conventional fractionated RT (50-50.4 Gy, 1.8-2.0 Gy per fraction).

S-1 is an orally administered chemotherapeutic drug that has been widely used in East Asia since several studies demonstrated promising effectiveness and safety in patients with digestive system tumors.^8,10,11,12,13,14^ A 2021 multicenter study^8^ demonstrated that conventional fractionated RT (54 Gy in 30 fractions) concurrent with oral S-1 could achieve superior overall survival (OS) compared with conventional fractionated RT alone.^8^ In this multicenter, phase III randomized clinical trial, we aimed to further assess the efficacy and toxicity of SIB-RT (59.92 Gy in 28 fractions in primary tumor and metastatic lymph nodes and 50.4 Gy in 28 fractions in the involved lymphatic drainage region) with concurrent and consolidated oral S-1 chemotherapy as a treatment approach for patients aged 70 years and older with inoperable ESCC.

## Methods

### Study Design

This multicenter, open-label, phase III randomized clinical trial was conducted between March 2017 and April 2020 in patients enrolled in 10 centers in China. The study was approved by the institutional review boards and independent ethics committees of the participating centers. The study was performed in accordance with the Declaration of Helsinki. Written informed consent was obtained from all participants. This study followed the Consolidated Standards of Reporting Trials (CONSORT) reporting guideline for randomized studies. The trial protocol and statistical analysis plan are available in Supplement 1.

### Patients

Patients were eligible for inclusion in this prospective multicenter phase III trial if they met the following criteria: age 70 years or older, histopathologically confirmed ESCC, clinical stage II to III or clinical stage IV disease with metastatic lymph nodes in the supraclavicular or celiac trunk area (according to the sixth edition of the American Joint Committee on Cancer classification^15^), Eastern Cooperative Oncology Group (ECOG) performance score (PS) of 1 or less, Charlson Comorbidity Index (CCI) of 3 or less, and hematopoietic, liver, and kidney function within reference ranges.

### Sample Size and Randomization

In this study, we used a superiority trial design. The probability of the outcome event in the test population was approximately 35%. It was expected to take 4 years to enroll all participants, with a follow-up period of 1 year after the last patient randomization. As such, a minimum of 134 patients was required for each group to achieve 80% power at the 5% level to detect a 10% increment in 1-year OS. Based on a 10% dropout rate, a final sample size of 150 for each group was required. Eligible patients were randomized 1:1 to receive S-1–based definitive SIB-RT followed by consolidated chemotherapy (CRTCT group) or SIB-RT alone (RT group) by a central randomization center (National Cancer Center, National Clinical Research Center for Cancer, Cancer Hospital, Beijing, China). Sequential assignment of patients was performed with R software using random block sizes of 4 with stratification of disease stage (IIa vs IIb vs III vs IVa vs IVb) and participating center. A random assignment number was allocated to each patient and provided to the respective investigators via telephone.

According to our protocol, when 80% of the patients (240 patients) enrolled in the study by February 19, 2019, 34 of 127 patients (26.8%) assigned to the CRTCT group were unable to complete concurrent chemotherapy and only 20 patients (15.7%) stopped treatment owing to treatment-related toxic effects. We subsequently enlarged the sample size by retaining the sample size of RT group according to the initial plan (150 patients) and changed the randomization proportion from 1:1 to 4:3 (CRTCT group:RT group) to enroll more patients into the CRTCT group. Consequently, the number of patients who completed RT in the RT group was similar to those who completed concurrent chemoradiotherapy in CRTCT group (133 patients vs 134 patients).

### Procedures

The enrolled patients were recommended to undergo nutritional interventions, including feeding tube or gastrostomy feeding before treatment if assessment of their nutritional status indicated a risk of malnutrition. The baseline imaging included endoscopy with biopsy, endoscopic ultrasonography, barium esophagography, chest and abdominal computed tomography (CT), cervical ultrasonography and CT, and, if available, positron-emission tomography (PET) CT. Comorbid conditions of enrolled participants were evaluated with the CCI, which has been shown to be an independent risk factor associated with surgical mortality as well as long-term survival.

In the CRTCT group, concurrent S-1 was administered orally twice daily on RT days (total daily dosage, 40, 50, or 60 mg/m^2^ based on body surface area). At 4 to 8 weeks after SIB-RT ended, consolidated S-1 administration continued at the same dosage. A cycle was 3 weeks, with S-1 administrated on days 1 to 14 in each cycle and a total of 4 cycles.

Radiation was delivered using IMRT or volumetric-modulated arc therapy. The gross tumor volume (GTV) was defined as visible primary tumor delineated by physicians using all possible resources (barium esophagram, CT, esophagogastroduodenoscopy, endoscopic ultrasonography, and if available, PET CT), metastatic regional nodes. The clinical target volume (CTV) consisted of the primary tumor plus a 0.6- to 0.8-cm circumferential margin, a 3-cm craniocaudal margin and metastatic regional nodes, plus a 0.5-cm margin in all directions and covering the corresponding lymphatic drainage region. The PTV is defined as CTV plus a uniform 0.5-cm margin. For SIB-RT, the PGTV was administered at 59.92 Gy (EQD2, 60.62Gy) and the PTV was administered at 50.4 Gy, in 28 fractions each.^9,10^

All patients will be followed-up for at least 5 years after completion of the protocol, every 3 months for the first 2 years, every 6 months for years 3 to 5, and once a year after 5 years. Every follow-up should include history-taking; bloodwork (basic metabolic panel); contrast-enhanced CT of the neck, thorax, and abdomen; ultrasonography of the neck and abdomen; upper gastrointestinal contrast; endoscopy (in patients experiencing recurrence or progression of dysphagia); bone scan (in patients experiencing bone pain or with elevated alkaline phosphatase levels); magnetic resonance imaging of the brain (in patients experiencing any symptoms related to central nervous system); and documentation of patients’ status of survival, disease progression, subsequent treatment, nutrition, quality of life, and late toxic effects.

### Outcomes

The primary end point was OS, and the secondary end points were progression-free survival (PFS), local-regional recurrence–free survival, distant metastasis–free survival, and treatment-related toxic effects. The time to each outcome was calculated from the date of randomization. All treatment-related toxic effects were graded according to the National Cancer Institute Common Terminology Criteria for Adverse Events, version 4.0.

### Statistical Analysis

Primary and secondary end points were evaluated in the intention-to-treat (ITT) population (all randomized patients). We used χ^2^ test to evaluate whether the missingness was at random or not at random. When the missingness was confirmed as at random, we conducted missingness imputation using weighted log-rank testing of the ITT population to simulate the condition in which the randomization proportion between RT group and CRTCT group was 1:1. Meanwhile, we applied post hoc analyses of generalized Wilcoxon test and Tarone-Ware test of the ITT population, post hoc survival analysis of the per-protocol (PP) population (randomized participants who completed treatment as planned), and post hoc subgroup analyses of patients with different general condition or cancer status. Statistical analysis was performed using the SPSS for Windows program, version 21.0 (IBM) and R version 3.4.1 (R Project for Statistical Computing). All statistical tests were 2-sided, and *P* < .05 was considered to indicate statistical significance. The analyses of data were completed on March 22, 2022.

## Results

### Patient Characteristics

**Figure 1.**
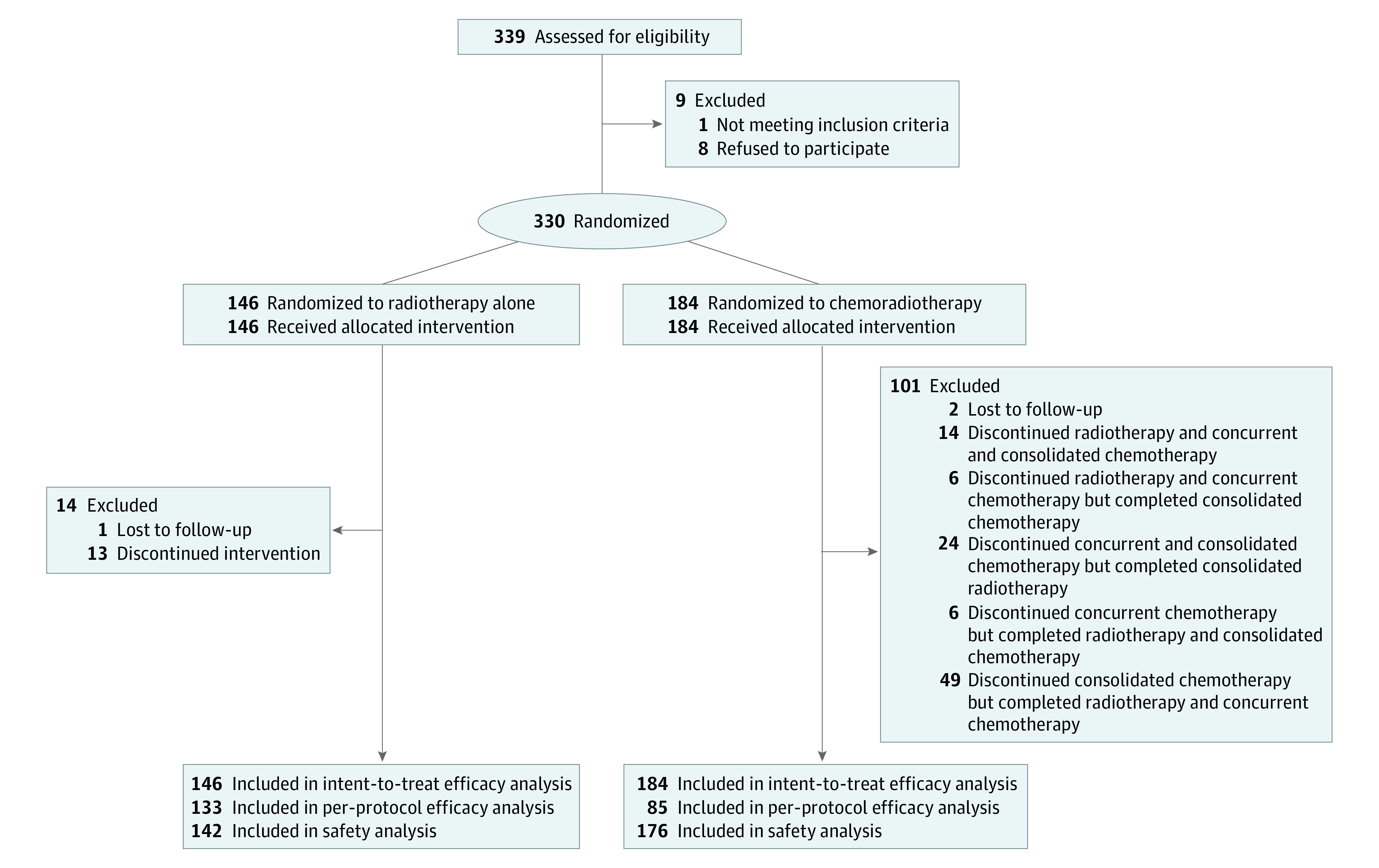
Patient Recruitment Flowchart

Between March 2017 and April 2020, 339 patients were screened in 10 centers in China. Finally, 330 patients were randomized (median [IQR] age, 75.5 [72-79] years; 220 [66.7%] male patients), with 184 patients in the CRTCT group and 146 patients in the RT group (Figure 1). There were 33 patients (22.6%) in the RT group and 39 patients (21.2%) in the CRTCT group aged 80 years or older. Most patients were assessed with a ECOG PS of 1 (137 patients [93.8%] in the RT group and 165 patients [89.7%] in the CRTCT group). Meanwhile, 107 patients (73.3%) in the RT group and 121 patients (67.9%) in the CRTCT group were diagnosed with stage III or IV ESCC. There were no marked differences in demographic characteristics, except more patients with lower CCIs were included in the RT group (Table 1). Causes of discontinuance of RT and concurrent and consolidated chemotherapy are listed in eTable 1 in Supplement 2.

**Table 1.** Baseline Characteristics of Included Patients

Characteristic	Patients, No. (%)	
	RT (n = 146)	CRTCT (n = 184)
Age, y		
<80	113 (77.4)	145 (78.8)
≥80	33 (22.6)	39 (21.2)
Median (IQR)	76 (73-79)	75 (72-79)
Sex		
Male	92 (63.0)	128 (69.6)
Female	54 (37.0)	56 (30.4)
ECOG performance score		
0	9 (6.2)	19 (10.3)
1	137 (93.8)	165 (89.7)
Charlson Comorbidity Index		
0	116 (79.5)	123 (66.8)
1	19 (13.0)	42 (22.8)
2	11 (7.5)	19 (10.3)
Mini nutritional assessment score		
<17	20 (13.7)	33 (17.9)
17-23.5	75 (51.4)	94 (51.1)
≥24	51 (34.9)	57 (31.0)
Median (IQR)	22 (19-24)	22 (17.6-24)
BMI		
<18.5	19 (13.0)	26 (14.1)
18.5-23.9	82 (56.2)	104 (56.5)
≥24	45 (30.8)	54 (29.3)
Median (IQR)	22.7 (19.7-24.7)	22.2 (20.3-24.4)
Pretreatment dietary intake		
General diet	27 (18.5)	18 (9.8)
Soft diet	49 (33.6)	65 (35.3)
Fluid diet	67 (45.9)	93 (50.5)
Aphagosis	3 (2.1)	8 (4.3)
Tobacco consumption		
No	87 (59.6)	112 (60.9)
Yes	59 (40.4)	72 (39.1)
Alcohol consumption		
No	118 (80.8)	158 (85.9)
Yes	28 (19.2)	26 (14.1)
Tumor location		
Upper third	44 (30.1)	71 (38.6)
Middle third	70 (47.9)	70 (38.0)
Lower third	32 (21.9)	43 (23.4)
Length, cm		
<5	64 (43.8)	64 (34.8)
≥5	82 (56.2)	120 (65.2)
Median (IQR)	5.0 (4.0-6.0)	5.0 (4.0-6.0)
T stage		
T1	4 (2.7)	2 (1.1)
T2	15 (10.3)	16 (8.7)
T3	87 (59.6)	124 (67.4)
T4	40 (27.4)	42 (22.8)
N stage		
N0	42 (28.8)	61 (33.2)
N1	104 (71.2)	123 (66.8)
M stage		
M0	127 (87.0)	163 (88.6)
M1a	9 (6.2)	14 (7.6)
M1b	10 (6.8)	7 (3.8)
TNM stage		
IIA	27 (18.5)	47 (25.5)
IIB	12 (8.2)	12 (6.5)
III	88 (60.3)	104 (56.5)
IVA	10 (6.8)	14 (7.6)
IVB	9 (6.2)	7 (3.8)
RT		
Incomplete	13 (8.9)	20 (10.9)
Completed	133 (91.1)	164 (89.1)
Concurrent chemotherapy		
Incomplete	NA	50 (27.2)
Completed	NA	134 (72.8)
Adjuvant chemotherapy		
Incomplete	NA	87 (47.3)
Completed	NA	97 (52.7)

Abbreviations: BMI, body mass index (calculated as weight in kilograms divided by height in meters squared); CRTCT, simultaneous integrated boost radiotherapy with concurrent and consolidated oral S-1 chemotherapy; NA, not applicable; RT, simultaneous integrated boost radiotherapy alone.

### Survival

**Figure 2.**
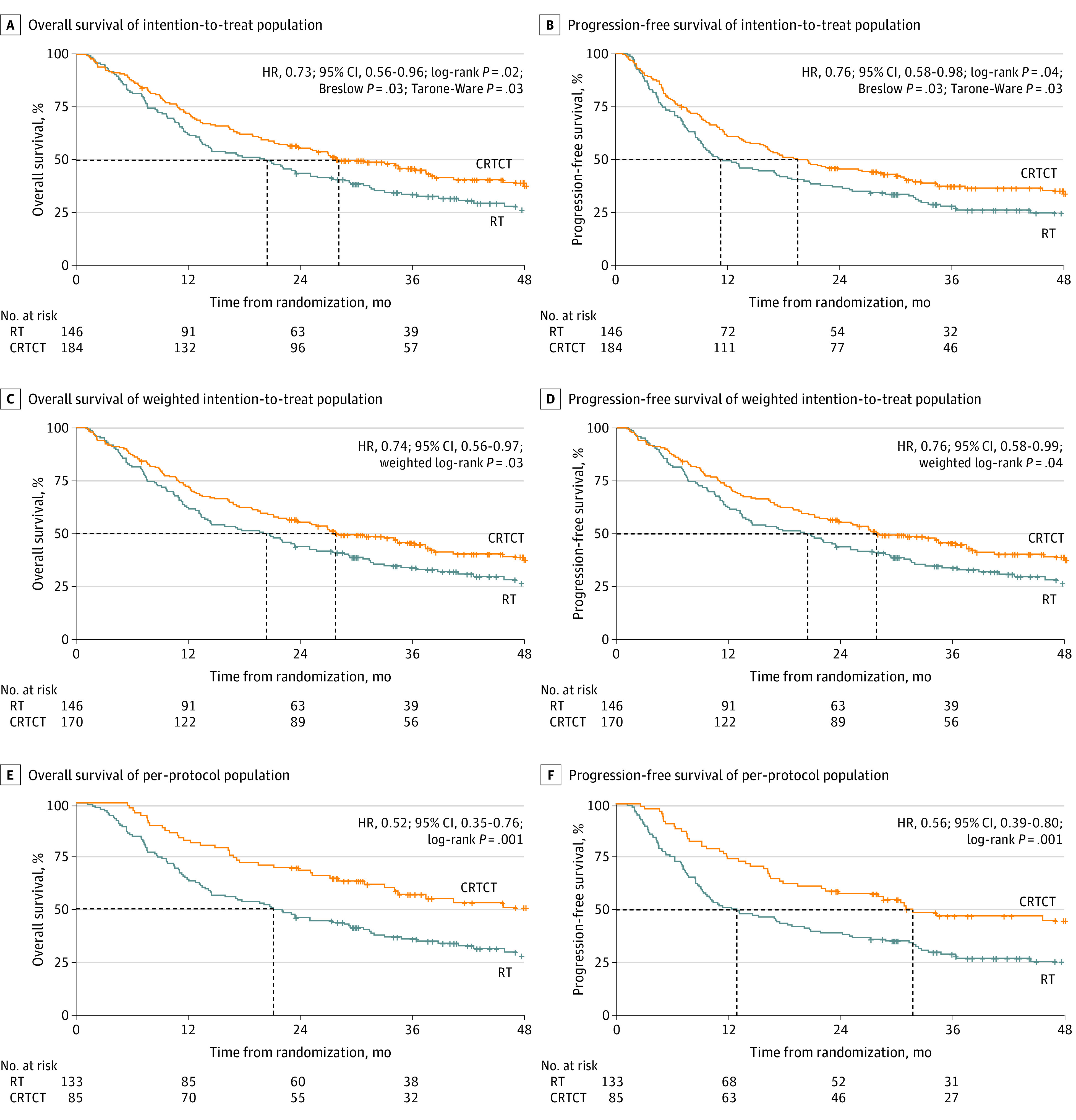
Survival Outcomes of the Intention-to-Treat, Weighted Intention-to-Treat, and Per-Protocol Populations CRTCT indicates simultaneous integrated boost radiotherapy with concurrent and consolidated oral S-1 chemotherapy; HR, hazard ratio; RT, simultaneous integrated boost radiotherapy alone.

At the time of analysis (March 22, 2022), the median (IQR) follow-up time for the surviving patients in the ITT population was 42.9 (38.8-47.0) months in the CRTCT group and 44.4 (39.3-49.4) months in the RT group. The median (IQR) OS was 28.1 (19.7-36.6) months in the CRTCT group and 20.1 (13.8-26.5) months in the RT group. The OS rates in the CRTCT group and RT group were 72.2% vs 62.3% at 1 year, 55.7% vs 43.8% at 2 years, and 46.2% vs 33.9% at 3 years (hazard ratio [HR], 0.73; 95% CI, 0.56-0.96; log-rank *P* = .02; Breslow *P* = .03; Tarone-Ware *P* = .03) (Figure 2A). The median (IQR) PFS was 19.5 (12.8-26.2) months in the CRTCT group and 11.1 (6.7-15.4) months in the RT group. The PFS rates in the CRTCT group and RT group were 60.8% vs 49.3% at 1 year, 45.4% vs 37.0% at 2 years, and 37.3% vs 27.9% at 3 years (HR, 0.76; 95% CI, 0.58-0.98; log-rank *P* = .04; Breslow *P* = .03; Tarone-Ware *P* = .03) (Figure 2B). As for the causes of death in both groups, 163 patients (79.5%) died of ESCC, 26 patients (12.7%) died of comorbidities, 9 patients (4.4%) died of treatment-related complications, 4 patients (2.0%) died from unintentional injuries, 1 patient (0.5%) died of a secondary primary tumor, and 2 patients (1.0%) died of unknown causes (eTable 2 in Supplement 2).

**Figure 3.**
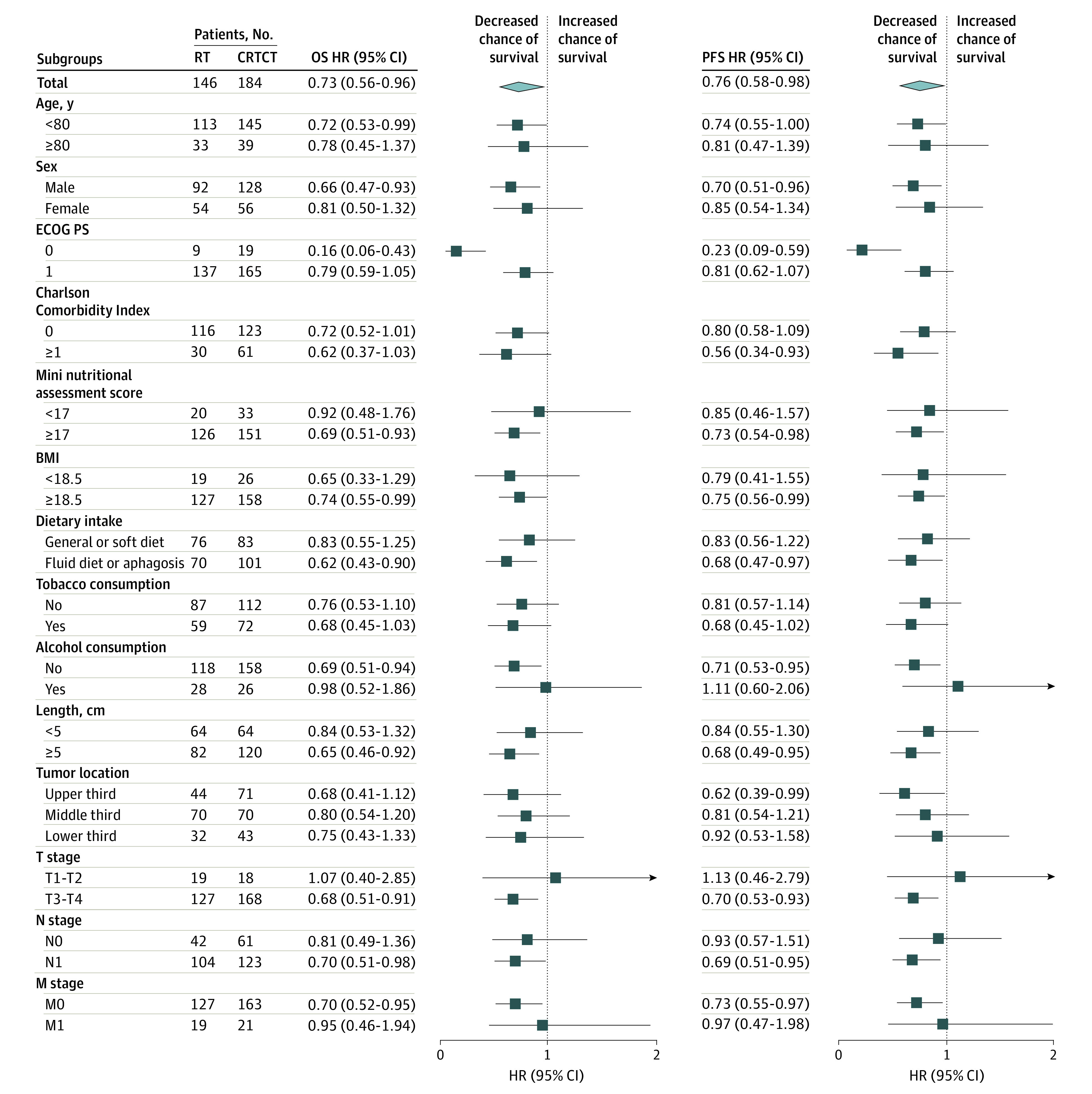
Subgroup Analyses of Overall Survival (OS) and Progression-Free Survival (PFS) in the Intention-to-Treat Population BMI indicates body mass index (calculated as weight in kilograms divided by height in meters squared); CRTCT, simultaneous integrated boost radiotherapy with concurrent and consolidated oral S-1 chemotherapy; ECOG PS, Eastern Cooperative Oncology Group performance score; HR, hazard ratio; RT, simultaneous integrated boost radiotherapy alone.

Subgroup analyses showed that different baseline characteristics (age, sex, ECOG PS, CCI, nutrition status, dietary status, tobacco or alcohol consumption status) or cancer status (tumor length, location or lymph node metastasis status) were associated with different outcomes with CRTCT in terms of OS, except for patients with T1-2 lesion (Figure 3). Likewise, different baseline characteristics (age, sex, ECOG PS, CCI, nutrition status, dietary status, and tobacco consumption status) and cancer statuses (tumor length, location or lymph nodes metastasis status) were associated with different outcomes from CRTCT in terms of PFS, except for patients with history of alcohol consumption or T1-2 lesion (Figure 3).

### Sensitivity Analysis

The demographic characteristics of the population before and after changing randomization ratios were not significantly different between CRTCT and RT groups, except there were more patients with primary lesions 5 cm or more in length in the CRTCT group (eTable 3 and eTable 4 in Supplement 2). Analysis using χ^2^ test showed the reasons for treatment discontinuity were not significantly different between the CRTCT group and the RT group (eTable 1 in Supplement 2); therefore, it could be assumed that the dropout from the 2 groups was at random. Weighted log-rank testing of the ITT population showed superior OS and PFS outcomes in the CRTCT group. The median (IQR) OS was 27.8 (22.6-38.4) months in the CRTCT group and 20.5 (13.8-27.5) months in the RT group. The OS rates in the CRTCT group and the RT group were 72.4% vs 62.3% at 1 year, 55.4% vs 43.8% at 2 years, and 45.7% vs 33.9% at 3 years (HR, 0.74; 95% CI, 0.56-0.97; weighted log-rank *P* = .03) (Figure 2C). The median (IQR) PFS was 19.5 (15.9-30.0) months in the CRTCT group and 11.3 (9.3-18.4) months in the RT group (HR, 0.76; 95% CI, 0.58-0.99; weighted log-rank *P* = .04) (Figure 2D).

In the PP population analysis, the median OS was not reached in CRTCT group, and the median (IQR) OS was 21.2 (14.3-28.0) months in the RT group. The OS rates in the CRTCT group and the RT group were 82.4% vs 63.9% at 1 year, 68.2% vs 45.9% at 2 years, and 56.8% vs 35.7% at 3 years (HR, 0.52; 95% CI, 0.35-0.76; log-rank *P* = .001) (Figure 2E). The median (IQR) PFS was 31.8 (14.5-49.1) months in CRTCT group and 13.0 (7.5-18.4) months in the RT group (HR, 0.56; 95% CI, 0.39-0.80; *P* = .001) (Figure 2F).

Among patients who completed concurrent chemotherapy in the CRTCT group, patients who completed consolidation with S-1 attained better survival rates compared with those who discontinued or refused consolidation (3-year OS: 56.8% vs 47.1%; log-rank *P* = .04; 3-year PFS: 45.0% vs 30.4%; log-rank *P* = 0.008) (eFigure in Supplement 2). Furthermore, the OS of patients who discontinued consolidation with S-1 due to treatment-related toxic effects or comorbidities was similar to those who discontinued consolidation with S-1 due to other causes (3-year OS: 40.6% vs 38.0%; log-rank *P* = .91).

### Treatment-Related Toxic Effects

A total of 318 patients were enrolled in safety analysis. There was no significant difference in the incidence of toxic effects higher than grade 3. Grade 5 toxic effects occurred in 5 patients in each group, including 1 patient who experienced myelosuppression and 4 patients with pneumonitis in the RT group and 3 patients with pneumonitis and 2 patients with fever in the CRTCT group (Table 2; eTable 5 in Supplement 2).

**Table 2.** Incidence of Adverse Events

Adverse event	Grade, No. (%)											
	RT (n = 142)						CRTCT (n = 176)					
	1	2	3	4	5	1		2	3	4	5
Anemia	43 (30.3)	12 (8.5)	0	0	1 (0.7)	70 (39.8)		20 (11.4)	2 (1.1)	0	0
Leukopenia	42 (29.6)	27 (19.0)	7 (4.9)	0	1 (0.7)	40 (22.7)		61 (34.7)	14 (8.0)	1 (0.6)	0
Neutropenia	28 (19.7)	7 (4.9)	4 (2.8)	0	1 (0.7)	33 (18.8)		26 (14.8)	8 (4.5)	1 (0.6)	0
Thrombocytopenia	15 (10.6)	7 (4.9)	0	0	0	34 (19.3)		10 (5.7)	1 (0.6)	1 (0.6)	0
Nausea	63 (44.4)	17 (12.0)	5 (3.5)	0	0	66 (37.5)		34 (19.3)	15 (8.5)	0	0
Vomiting	21 (14.8)	10 (7.0)	1 (0.7)	0	0	35 (19.9)		15 (8.5)	5 (2.8)	1 (0.6)	0
Fatigue	85 (59.9)	13 (9.2)	1 (0.7)	0	0	96 (54.5)		23 (13.1)	5 (2.8)	0	0
Diarrhea	10 (7.0)	0	0	0	0	8 (4.5)		2 (1.1)	1 (0.6)	0	0
Esophagitis	27 (19.0)	69 (48.6)	8 (5.6)	0	0	23 (13.1)		91 (51.7)	21 (11.9)	0	0
Pneumonitis	16 (11.3)	8 (5.6)	3 (2.1)	0	3 (2.1)	25 (14.2)		13 (7.4)	5 (2.8)	1 (0.6)	3 (1.7)
Fever	11 (7.7)	2 (1.4)	0	0	0	19 (10.8)		5 (2.8)	2 (1.1)	0	2 (1.1)
Radiodermatitis	66 (46.5)	6 (4.2)	0	0	0	79 (44.9)		6 (3.4)	0	0	0
Weight loss	41 (28.9)	3 (2.1)	1 (0.7)	0	0	49 (27.8)		4 (2.3)	1 (0.6)	0	0
Hypoalbuminemia	26 (18.3)	3 (2.1)	0	0	0	44 (25.0)		11 (6.3)	0	0	0
Transaminitis	1 (0.7)	0	0	0	0	6 (3.4)		0	2 (1.1)	0	0

Abbreviations: CRTCT, simultaneous integrated boost radiotherapy with concurrent and consolidated oral S-1 chemotherapy; RT, simultaneous integrated boost radiotherapy alone.

### Failure Pattern

Disease progression was observed in 180 patients. Among patients with disease progression, 108 (60.0%) had recurrence in the esophagus, 25 (13.5%) had recurrence in the regional lymph nodes, and 75 (41.4%) had recurrence in the distant lymph nodes or organs. The local-regional recurrence–free survival rates in the CRTCT group and the RT group were 62.9% vs 54.8% at 1 year, 50.3% vs 39.7% at 2 years, and 41.3% vs 29.3% at 3 years (HR, 0.75; 95% CI, 0.57-0.97; log-rank *P* = .03). The distant metastasis–free survival rates in the CRTCT group and the RT group were 69.0% vs 54.8% at 1 year, 48.7% vs 40.3% at 2 years, and 41.2% vs 32.3% at 3 years (HR, 0.76; 95% CI, 0.58-1.00; log-rank *P* = .50).

## Discussion

This phase III randomized clinical trial assessed the effectiveness of SIB-RT with concurrent and consolidated chemotherapy in patients aged 70 years and older with ESCC. The findings suggest that patients treated with SIB-RT combined with oral S-1 chemotherapy experienced significant improvement in OS and PFS compared with SIB-RT alone and acceptable treatment-related toxic effects, indicating that SIB-RT with oral S-1 chemotherapy could be an appropriate treatment approach for patients aged 70 years and older.

Patients tend to tolerate intravenous chemotherapy less well with age, and patients aged 70 years and older are at increased risk of treatment-related toxic effects. A 2011 multicenter study found that when treated with intravenous chemotherapy, more than 50% of patients older than 65 years encountered at least 1 grade 3 to 5 toxic effect and 2% died as result of treatment-related toxic effects.^4^ In a study of patients aged 70 years or older with ESCC treated with intravenous chemotherapy with or without radiotherapy, a significantly higher proportion of patients experienced severe treatment-related leucopenia (70% of patients) and anemia (51.5% of patients) compared with younger patients, resulting in more than 50% of elderly patients discontinuing concurrent chemoradiotherapy and inferior survival outcomes.^3,16^ S-1 is an orally administered chemotherapeutic drug that has been widely used in East Asia since several studies demonstrated promising effectiveness and safety in patients with digestive system tumors.^8,10,11,12,13,14^ In our previous multicenter phase II prospective trial, SIB-RT with concurrent and consolidated oral S-1 chemotherapy yielded satisfactory tumor response rates and manageable toxic effects in older patients with ESCC.^10^

In this study, we observed an improvement of OS in the CRTCT group through ITT analysis. Sensitivity analyses also showed significant survival benefit in the CRTCT group. In addition, the incidence of severe toxic effects in the CRTCT group was not significantly increased compared with that in the RT group. Analogously, a 2021 phase III randomized clinical trial conducted by Ji et al^8^ showed definitive RT concurrent with S-1 provided significant benefits over RT alone in older patients (2-year OS: 53.2% vs 35.8%; *P* = .002). In that study,^8^ the 2-year OS of patients treated with RT and concurrent S-1 was similar to that of patients who completed concurrent chemotherapy in our study (53.2% vs 54.3%) and lower than that of patients treated with RT concurrent with single- or double-agent intravenous chemotherapy in a 2019 multicenter retrospective study (59.0% and 57.0%, respectively).^17^ Additional consolidated therapy with RT is a potential approach to reduce the risk of disease recurrence and death for some patients.^18,19^ Furthermore, subgroup analysis in this study indicated that patients with T3-4 or N1 lesion were more likely to benefit from CRTCT. Hence, for older patients with locally advanced disease, intensifying the treatment through adding S-1 chemotherapy to SIB-RT could potentially result in more favorable survival outcomes.

Based on our previous prospective phase I/II dose escalation study of SIB-RT concurrent with weekly chemotherapy of paclitaxel and nedaplatin as chemoradiotherapy for patients younger than 70 years with locally advanced ESCC, we recommended boosting the dose to the PGTV to 59.92 Gy (EQD2, 60.62 Gy), with a standard dose of 50.4 Gy to the PTV.^9^ Analogously, several previous studies found that patients receiving chemoradiotherapy with SIB to the primary tumor and involved lymph nodes with a radiation dose of more than 60 Gy attained superior 2-year OS and local-regional control compared with contemporaneous institutional cohorts receiving standard-dose chemoradiotherapy and that treatment-related toxic effects were within acceptable bounds.^20,21,22^ In this phase III study, this dose achieved similar clinical efficacy, with acceptable toxic effects. In the era of IMRT, the ARTDECO study^23^ showed that patients treated with radiation dose-escalation up to 61.6 Gy administered to the primary tumor using the SIB-RT approach had OS similar to those treated with a standard radiation dose of 50.4 Gy. Although the survival benefit of SIB-RT is controversial, considering SIB-RT can shorten the treatment course, avoid resimulation and replanning in the middle of the treatment course, increase cost-effectiveness, and allow more precise evaluation of radiotherapy plans,^24^ SIB-IMRT still appears to be equivalent to conventional-fraction IMRT in treatment efficacy and safety and could become an alternative option for definitive RT of ESCC. Furthermore, grades 4 and 5 toxic effects occurred in very few patients in the CRTCT group of our study. Therefore, based on effectiveness and safety considerations, we recommend involved-field RT with a SIB radiation dose of 59.92 Gy or 50.4 Gy in 28 fractions combined with well-tolerated concurrent and consolidated oral S-1 chemotherapy as an optional approach for patients aged 70 years and older with ESCC.

### Limitations

This study has some limitations. First, all patients in this study had squamous cell carcinoma, so the results may not be generalizable to adenocarcinoma. In addition, for financial reasons, PET CT is not part of the standard examination in China and was applied to patients only if it was available. Furthermore, due to poor adherence among included patients and the outbreak of the COVID-19 pandemic, 46 of 184 patients (25.0%) in the CRTCT group did not complete the entire course of chemotherapy, whereas only 9 patients (4.9%) in the CRTCT group died or relapsed before completing chemotherapy.

## Conclusions

In this randomized clinical trial of patients aged 70 years and older with ESCC treated with SIB-RT with or without concurrent and consolidated oral S-1 chemotherapy, the combination of SIB-RT technique with concurrent and consolidated oral S-1 chemotherapy yielded improvements in OS and PFS for patients aged 70 years and older with ESCC. SIB-RT with oral S-1 chemotherapy should be considered as an option for definitive treatment approaches for patients aged 70 years and older with inoperable ESCC, as it improved survival outcomes without additional treatment-related toxic effects compared with SIB-RT alone.

## Notes

Supplement 1.
Trial Protocol and Statistical Analysis Plan

Supplement 2. **eTable 1.** Causes of Treatment Discontinuity in RT and CRTCT Groups **eTable 2.** Causes of Death in RT and CRTCT Groups **eTable 3.** Baseline Characteristics of Patients Enrolled Before February 19, 2019 **eTable 4.** Baseline Characteristics of Patients Enrolled After February 19, 2019 **eTable 5.** Incidence of Severe Adverse Events (Grade 3-5) **eFigure.** Overall Survival and Progression-Free Survival of Patients Who Completed Concurrent Chemotherapy With or Without Consolidated Chemotherapy

Supplement 3.
Members of the Jing-Jin-Ji Esophageal and Esophagogastric Cancer Radiotherapy Oncology Group (3JECROG)

Supplement 4.
Data Sharing Statement
