# Supplementary material for: Effectiveness of S-1–Based Chemoradiotherapy in Patients 70 Years and Older With Esophageal Squamous Cell Carcinoma: A Randomized Clinical Trial
Source: JAMA Netw Open. 2023 May 17;6(5):e2312625. doi: 10.1001/jamanetworkopen.2023.12625 (PMC10193185; doi:10.1001/jamanetworkopen.2023.12625)
Supplement: Supplement 1. — Trial Protocol and Statistical Analysis Plan [file jamanetwopen-e2312625-s001.pdf]

A Phase 3, Multicenter, Prospective Study of Tegafur-Gimeracil-Oteracil (S-1) with Simultaneous-integrated Boost Radiotherapy Versus Simultaneous-integrated Boost Radiotherapy Alone in Geriatric Esophageal Cancer/Esophagogastric Junction Cancer (3JECROG-P01)

Investigator's Brochure

Principal Leads	Xiao Zefen, Han Chun, Pang Qingsong and Zhao Yidian
Cooperative Group	Fan-Jingjinyi Multicenter Cooperative Group of Esophageal Tumors
Start Date	March 2017
End Date	April 2021
Leading Site	Cancer Hospital, Chinese Academy of Medical Sciences
Participating Sites	Tianjin Medical University Cancer Institute & Hospital<br>Anyang Cancer Hospital, Henan Province<br>Affiliated Hospital of Hebei Medical University<br>Tengzhou People's Hospital, Shandong Province<br>Jiangsu Province Hospital<br>Fujian Cancer Hospital<br>First Affiliated Hospital of Zhengzhou University<br>First Affiliated Hospital of Xinxiang Medical University, Henan Province

Study Overview

I. Study Rationale

1. Lack of Therapeutic Guidelines for Geriatric Esophageal and Esophagogastric Junction Cancer

No prospective clinical studies exist for geriatric esophageal and esophagogastric cancer that have a large sample size and a high-level evidence to support the U.S. National Comprehensive Cancer Network (NCCN), the European Society for Medical Oncology (ESMO), or the Chinese treatment guidelines, resulting in a lack of recommended treatment options. Clinically, the concurrent chemoradiotherapy regime for esophageal cancer in patients aged 70 years or younger is still widely used clinically with older patients. However, this regime does not address the clinical characteristics of older patients who may poorly tolerate the highly intensive treatment and who have a high incidence of concomitant complications. As a result, it is difficult to complete the treatment. Therefore, the need is urgent for a reasonable and effective treatment regime specifically for geriatric esophageal and esophagogastric cancer.

2. High Mortality and Poor Therapeutic Outcomes for Esophageal Cancer

In China, esophagogastric cancer is a malignancy with a high incidence and poor prognosis, and its incidence and mortality account for 50% of the global rates of the disease[1]. Among patients with this cancer, those aged 70 years or older account for a high percentage of cases, between 30%–40%[2], and this rate is still growing in our increasingly aging society. Because older patients may not tolerate surgery well and may have multiple comorbidities, radiation therapy may be better accepted and be an effective treatment for geriatric esophagogastric cancer. However, RTOG8501[3], a prospective randomized study conducted in 1999 in the United States, found that radiotherapy alone was associated with a significantly lower survival rate than was concurrent chemoradiotherapy (the 5-year survival rates were 0% and 27%, respectively). Therefore, the NCCN treatment guidance recommends concurrent chemoradiotherapy. However, this study was based on standard radiotherapy, and no prospective studies have been reported for large patient cohorts since advanced radiotherapy techniques have been

adopted. Therefore, new research evidence, especially from studies of advanced radiotherapy techniques, is needed.

3. A Radiation Dose for Both Prevention and Treatment Area Achieved with Advanced Radiotherapy Techniques

With advances in the three-dimensional conformal radiation therapy (including 3D conformal radiation therapy [CRT] and inverse intensity-modulated radiation therapy [IMRT]), a Phase 2 clinical study of radical simultaneous-integrated boost (SIB)-IMRT with concurrent chemotherapy for esophageal cancer used a similar radiation dose to that used in the high-dose group in RTOG 94-05[4] and showed a mean survival rate of 23 months and a 3-year overall survival rate of 44.4%, a significant increase as compared to standard radiotherapy. This result supports the potential efficacy of SIB. However, whether SIB-IMRT can clinically gain wide application largely depends on its safety, and the challenging complications of SIB with concurrent chemotherapy, such as esophageal perforation, bleeding, and strictures, deserve close attention. Therefore, prospective Phase 3 studies with large patient samples are needed before the technique can be used clinically. However, such clinical data based on large samples for SIB-IMRT is still lacking.

4. Difficulties for Older Patients to Complete the Standard of Care Recommended by NCCN Because of Toxic and Side Effects

According to the U.S. NCCN and European ESMO treatment guidelines, the concurrent 5FU or capecitabine plus platinum-based two-drug chemoradiotherapy is recommended for patients with middle- to advanced-stage or inoperable esophageal cancer as the comprehensive treatment regime. However, the toxic and side effects of this regime are high. For example, the incidence ranges from 25% to 49% for Grade 3 adverse reactions, 6% to 21% for Grade 4 adverse reactions, and 2% to 9% for Grade 5 adverse reactions. No treatment guidelines exist that are dedicated to geriatric esophageal or esophagogastric junction cancer, except for some retrospective small-sample analyses.

For example, Tougeron et al. retrospectively analyzed the data from 109 patients with esophageal cancer aged 70 years or older who showed a clinical complete response (cCR) rate of 57.8% after receiving concurrent chemoradiotherapy consisting of cisplatin plus 5FU or cisplatin plus irinotecan, while multivariate analysis showed that concurrent

chemoradiotherapy, a radiation dose at 80% or more of the planned dose, and the Charlson score were all independent predictive factors for prognosis. However, the incidence of adverse reactions of Grade 3 or more was also high, reaching 23.8% and leading to 15.6% of the patients being unable to complete treatment. In this study, the authors also found that the Charlson score was significantly associated with the patients' tolerance of the treatment.[5]

Therefore, the treatment modality of two-drug chemotherapy with concurrent radiotherapy has not been applied widely in clinical settings for geriatric cancer, especially in China, because of poor patient tolerance and a low treatment completion rate[6, 7]. Most hospitals use the conservative treatment regime of radiation only, although it is less effective than concurrent chemoradiotherapy. Therefore, will the advanced SIB-IMRT techniques combined with concurrent S-1 become a standard of care for geriatric esophageal and esophagogastric cancer? Insufficient evidence exists from prospective studies to answer this question.

5. Comprehensive Geriatric Assessment

Assessment of patients' quality of life (QoL) was largely ignored in prior prospective clinical studies in China; in addition, a Chinese-originated assessment system does not exist. Introduction of the internationally established standard evaluations to this study will enable a more humanized approach, with a focus on the observations of older patients' QoL. In fact, no published studies related to comprehensive geriatric assessment (CGA) have involved geriatric esophageal cancer.

CGA is a diagnostic process involving multi-lateral and multi-dimensional evaluation of an older patient's medical, mental, and physical functions, and on the basis of these results, a management plan is recommended to maintain or improve the functions to maximize the improvement or retention of QoL. CGA has been shown to be associated with a reduced incidence of treatment-related complications, improved QoL and physical functions, and reduced risk for hospitalization for some geriatric patients with tumors.[8]

Today it is important to choose a treatment regime by evaluating the potential for severe adverse reactions before the initiation of treatment. Corre *et al.* randomized 494 patients aged 70 or older who had non-small cell lung cancer (NSCLC) into a CGA (test)

group and an age-PS score (control) group to receive the different treatment plans designed. Those patients with a favorable CGA score in the test group and those younger than age 75 and generally in good medical health in the control group were both treated with the carboplatin-based two-drug chemotherapy; those with a poor CGA score in the test group received only the best supportive care, and those of advanced age in the control group received only chemotherapy with docetaxel. The study concluded that CGA was not associated with the prognosis of geriatric NSCLC but found that CGA reduced the incidence of treatment-related toxic reactions.[9]

Therefore, this current study will perform the CGA on patients with geriatric esophageal cancer before and after treatment, including evaluation of social support, general condition, physical functions, nutritional status, mental health, and cognitive capability. The role of CGA in guiding the customized treatment also will be explored by analyzing the relationship of the CGA evaluation results with the patients' survival rate, seriousness of adverse reactions, and QoL.

In summary, this research project is planned to be a prospective, multicenter (facilitated by the applicant's identity as the Fan-Jingjinyi Cooperative Group of Esophageal Cancer) Phase 3 clinical study. This study is expected to generate high-level research evidence to provide crucial data and rationales for treatment solutions for geriatric esophageal/esophagogastric junction cancer.

References

[1] Chen W, Zheng R, Baade PD, et al. Cancer statistics in China, 2015[J]. CA Cancer J Clin, 2016,66(2):115-132. DOI: 10.3322/caac.21338.

[2] Wu M, Van't VP, Zhang ZF, et al. A large proportion of esophageal cancer cases and the incidence difference between regions are attributable to lifestyle risk factors in China[J]. Cancer Lett, 2011,308(2):189-196. DOI: 10.1016/j.canlet.2011.05.003.

[3] Cooper JS, Guo MD, Herskovic A, et al. Chemoradiotherapy of locally advanced esophageal cancer: long-term follow-up of a prospective randomized trial (RTOG 85-01). Radiation Therapy Oncology Group[J]. JAMA, 1999,281(17):1623-1627.

[4] Minsky BD, Pajak TF, Ginsberg RJ, et al. INT 0123 (Radiation Therapy Oncology Group 94-05) phase III trial of combined-modality therapy for esophageal

cancer: high-dose versus standard-dose radiation therapy[J]. J Clin Oncol, 2002,20(5):1167-1174.
[5] Sasako M, Sakuramoto S, Katai H, et al. Five-year outcomes of a randomized phase III trial comparing adjuvant chemotherapy with S-1 versus surgery alone in stage II or III gastric cancer[J]. J Clin Oncol, 2011,29(33):4387-4393. DOI: 10.1200/JCO.2011.36.5908.
[6] Fukushima M, Sakamoto K, Sakata M, et al. Gimeracil, a component of S-1, may enhance the antitumor activity of X-ray irradiation in human cancer xenograft models in vivo[J]. Oncol Rep, 2010,24(5):1307-1313.
[7] Huang J, Cao Y, Wu L, et al. S-1-based therapy versus 5-FU-based therapy in advanced gastric cancer: a meta-analysis[J]. Med Oncol, 2011,28(4):1004-1011. DOI: 10.1007/s12032-010-9594-0.
[8] Cohen HJ, Feussner JR, Weinberger M, et al. A controlled trial of inpatient and outpatient geriatric evaluation and management[J]. N Engl J Med, 2002,346(12):905-912. DOI: 10.1056/NEJMsa010285.
[9] Corre R, Greillier L, Le CH, et al. Use of a Comprehensive Geriatric Assessment for the Management of Elderly Patients With Advanced Non-Small-Cell Lung Cancer: The Phase III Randomized ESOGIA-GFPC-GECP 08-02 Study[J]. J Clin Oncol, 2016,34(13):1476-1483. DOI: 10.1200/JCO.2015.63.5839.

Study Content

I. Study Objectives

To provide high-level clinical evidence for treatment recommendations for geriatric esophageal cancer and esophagogastric junction cancer by comparing the overall survival between patients receiving S-1 with concurrent SIB-IMRT and SIB-IMRT alone in the phase 3 RCT.

II. Study Protocol

1. **Study Design:** This study is an open, multicenter Phase III clinical trial. Approximately 15 participating centers throughout China are involved. The technique of SIB is adopted in this study with a dose of 50.4Gy/2.14Gy/28f to planning target volume (PTV) and 59.92Gy/2.14Gy/28f to planning gross tumor

volume (PGTV). S-1 is given both concurrent with and after radiotherapy. Patients enrolled are stratified by disease stage and study site and assigned to either SIB +S-1 group or SIB group using a 1:1 allocation ratio at randomization.

2. Calculation of Sample Size and Method of Randomization:

In the phase 3 study, we used a superiority trial design. The probability of the outcome event in this study's test population was approximately 35%. It was expected to take 4 years to enroll all subjects, with a follow-up period of 1 year after the last patient randomization. As such, a minimum of 134 patients was required for each group to achieve 80% power at the 5% level to detect a 10% increment in 1-year OS. Based on a 10% dropout rate, a final sample size of 150 for each arm was required. Eligible patients were randomized 1:1 to receive S-1-based definitive SIB-RT followed by consolidated chemotherapy (CRT-CT arm) or SIB-RT alone (RT arm) by a central randomization center (National Cancer Center/National Clinical Research Center for Cancer/Cancer Hospital, Beijing, China). Sequential assignment of patients was performed with R software using random block sizes of four with stratification of disease stage (IIa vs. IIb vs. III vs. IVa vs. IVb) and participating centers. A random assignment number was allocated to each patient and provided to the respective investigators via telephone. At the time point when 80% of the patients (240 patients) have been enrolled, the treatment competition and the cause of treatment discontinuity are intended to be analyzed. The enrollment of patients could proceed as the previous plan if the treatment completion rate is higher than 75%. In case of the treatment completion rate is lower than 75%, the cause of treatment discontinuity should be analyzed. If the incidence of treatment-related toxicities induced treatment discontinuity lower than 20%, the enrollment of patients would continue after changing the randomization ratio based on the incidence of incompletion to make the number of patients completed radiotherapy in the RT arm was similar to those completed concurrent chemoradiotherapy in the CRT-CT arm.

3. Study Subjects

1) Inclusion Criteria

- Either gender aged ≥70 years;
- Naïve esophageal and esophagogastric cancer, with a clinical stage of T1bN + M0-1a or T2-4N0-1M0-1a per AJCC/UICC 2002 (*i.e.*, Stages IIa–IVa);
- KPS score ≥70 or ECOG score of 0–1 and Charlson Comorbidity score ≤3;
- Squamous carcinoma or adenocarcinoma that is cytologically or pathologically confirmed;
- Epicenter of the tumor in the esophagogastric junction adenocarcinoma no more than 2 cm below the dentate line (*i.e.*, Siewert Types I/II);
- Clinical remittance of other malignant tumors (excluding curable non-melanoma skin cancer and cervical carcinoma in situ), if any, in more than 5 years;
- Laboratory parameters met as follows:

  Hemoglobin ≥100g/L; white blood cells (WBCs) ≥ 3.5×10^9/L; neutrophils ≥ 1.5×10^9/L; platelets ≥ 100×10^9/L; creatinine ≤ 1.0x upper normal limit (UNL); blood urea nitrogen (BUN) ≤ 1.0x UNL; alanine transaminase (ALT) and aspartate aminotransferase (AST) ≤ 1.5x UNL, alkaline phosphatase (ALP) ≤ 1.5x UNL, and total bilirubin (TBIL) ≤ 1.5x UNL;
- No history of allergy to 5-FU drugs; and
- No surgical, radiation, chemotherapy or any other anti-tumor therapies before enrollment; no prior radiation at the site to be treated with radiotherapy.

2) Exclusion Criteria

- History of malignant tumors at other sites within 5 years before enrollment, excluding curable non-melanoma skin cancer and cervical carcinoma in situ;
- Distant and hematogenous metastases beyond the supraclavicular lymph node area at diagnosis, including metastases in multiple retroperitoneal lymph nodes, bone, brain, lung, and liver and pleural fluid and ascites (with or without cancerous cells detected in the ascites);
- Inability to receive 3D CRT or IMRT;
- Prior radiation at the site to be radiated;
- Allergy to 5-FU drugs;

- Apparent abnormalities in hematology or liver and renal functions at clinical examination;
- Current obvious signs of perforation and deep ulcer of the esophagus (imaging confirmed);
- Presence of fistula, perforation, and cachexy in the esophagus;
- Current active infections such as active tuberculosis and hepatitis;
- Other severe diseases, such as myocardial infarction and cerebral infarction within 6 months or severe cardiac insufficiency and arrhythmia; mental disorders, including inability to cooperate; uncontrollable hypertension; and diabetes;
- Participation in other clinical trials currently or within 4 weeks before enrollment; and
- Other situations in which auditors find adequate reasons in registered studies to disqualify the patients, such as potential noncompliance with the protocol.

3) **Withdrawal and/or Termination Criteria:** Disease progression, voluntary patient withdrawal, or force majeure.

4) **Dropout Criteria:** Poor compliance such that the patients cannot complete the study according to the protocol; those who are lost during follow-up; and any other situation in which study staff deem it necessary to withdraw a subject.

4. Study Methods

1) Primary Study Objectives

To compare the overall survival between S-1 with concurrent SIB-IMRT (test group) and SIB-IMRT alone (control group).

2) Secondary Study Objectives

A. Progression-free survival;

B. Incidence of adverse reactions.

3) Stipulations for Positioning Simulation, Prescribed Dose, and Area of Radiation Therapy (Target Area)

A. Requirements for CT Positioning Simulation of Radiotherapy

CT Positioning Simulation: Patients with cancer in the lower part of the esophagus or in the esophagogastric junction will fast for 3–4 hours before

CT positioning simulation to reduce the volume difference in radiation due to gastric filling and will have a semi-liquid diet of 200–300 ml (quantified each time) 15 minutes before each CT scan and radiation. This step is not prescribed for patients with cancer in the upper or middle segment of the esophagus. All patients will be scanned in the supine position, with the arms stretched on the sides of the body. Those patients with tumors in the cervical or the upper segment of the esophagus are recommended to use an immobilization mask for the head, neck, and shoulders, and those with tumors in the middle or lower segment of the esophagus or in the esophagogastric junction are immobilized with a body membrane. Contrast-enhanced venography shall be performed, with a slice thickness of 0.5 cm. Patients who are allergic to the contrast may be scanned without enhancement.

B. Prescribed Dose

The SIB-IMRT technology will be used for patients in both groups, with 5 radiations per week.

a) Squamous carcinoma in any segment of the esophagus, or the less common esophageal adenocarcinoma

   95%PTV 50.4Gy/1.8Gy/28 times + SIB-PGTV 59.92Gy/2.14Gy/28 times

b) Squamous carcinoma and adenocarcinoma in the esophagogastric junction (Siewert Types I/II)

   95%PTV 45Gy/1.8Gy/25 times + SIB-PGTV 53.5Gy/2.14Gy/25 times

C. Definition and Outlining of the Target Area (Same for Both Groups)

a) *GTV:* Outline the primary tumor using the combined imaging results of the thoracic CT, upper gastrointestinal radiography, gastroscopy and intracavitary ultrasound, PET-CT, and nuclear MRI.

b) *GTVnd:* Metastatic or highly suspected metastatic lymph nodes suggested by imaging results.

c) *CTV:* A margin of 0.6–0.8 cm on the left, right, anterior, and posterior sides (all 4 sides) of the GTV and GTVnd (adjusted for anatomical barriers if encountered in expansion) and a margin of 3–5 cm in the upper and lower directions of the GTV. Specifically,

- *Cervical/Upper Segment:* Level 1 or the lower cervical part, bilateral supraclavicular areas, 2, 3P, and Levels 4 and 7.
  - ✓ *Upper limit:* 3–5 cm above the GTV or 1–1.5 cm above the metastatic lymph node area, whichever is higher.
  - ✓ *Lower limit:* 3 cm below the GTV or 1–1.5 cm below the metastatic lymph node area, whichever is lower.
- *Middle Segment:* Including 2, 3P, and Levels 4, 7, and 8. (*Note:* The supraclavicular area is not included.)
  - ✓ *Upper limit:* 3–5 cm above the GTV or 1–1.5 cm above the metastatic lymph node area, whichever is higher.
  - ✓ *Lower limit:* 3 cm below the GTV or 1– 1.5 cm below the metastatic lymph node area, whichever is lower.
- *Lower Segment/Siewert Types I/II:* Including Level 8, the cardia, and the left stomach and excluding the lymphatic drainage area in the truncus celiac.
  - ✓ *Upper limit:* 3–5 cm above the GTV or 1–1.5 cm above the metastatic lymph node area, whichever is higher.
  - ✓ *Lower limit*: 3 cm below the GTV or 1–1.5 cm below the metastatic lymph node area, whichever is lower. If the tumor invades the cardia, fundus of the stomach, or stomach curvature, the CTV is expanded to a margin of 1–2 cm below the GTV along the stomach wall and 1 cm along the stomach cavity.

d) *PGTV:* Including GTV + GTVnd, with a margin of 0.5 cm on the left, right, anterior, and posterior sides. If the upper/lower limit is GTV, PGTV is expanded to 1.0 cm above/below GTV. If the upper/lower limit is GTVnd, PGTV is expanded to 0.5 cm above/below GTVnd.

e) PTV is expanded to 0.5 cm of CTV in the 3 dimensions.

Note (see the figures below): **Outlining principles in the supraclavicular area: A. The outer area where no supraclavicular lymph node metastasis exists is the area within the internal carotid sheath. B. Expansion touches the outer edge of the jugular vein or the entire supraclavicular area dependent on the supraclavicular lymph node metastasis. See Figures A (Patient 1, without supraclavicular lymph node metastasis), B1−B3 (Patient 2, with oligo supraclavicular lymph node metastasis attached with internal carotid vein) and C1−C2 (Patient 3, with multiple lymph node metastasis in supraclavicular fossa).**

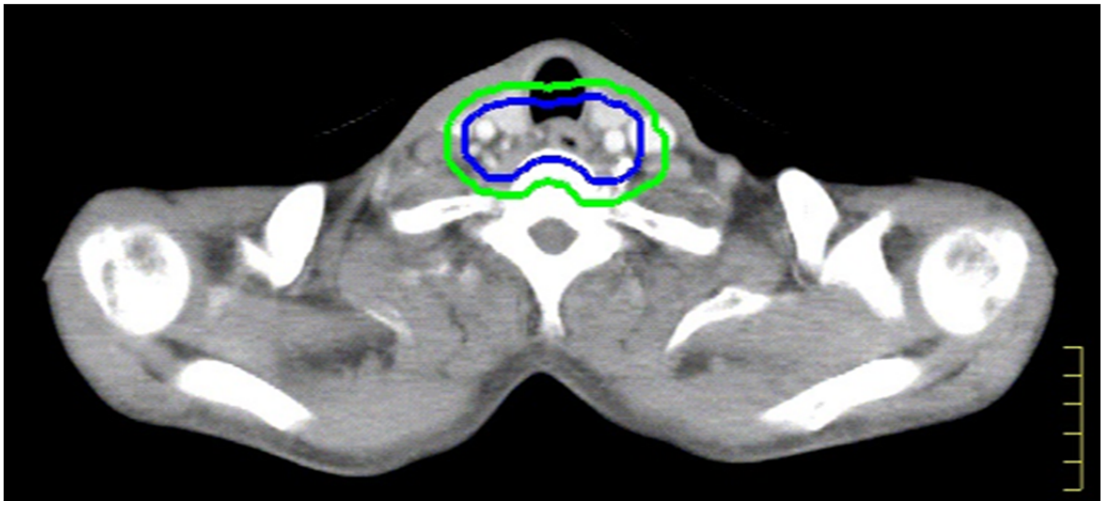

Figure A. Prevention area in the upper segment of the esophagus.

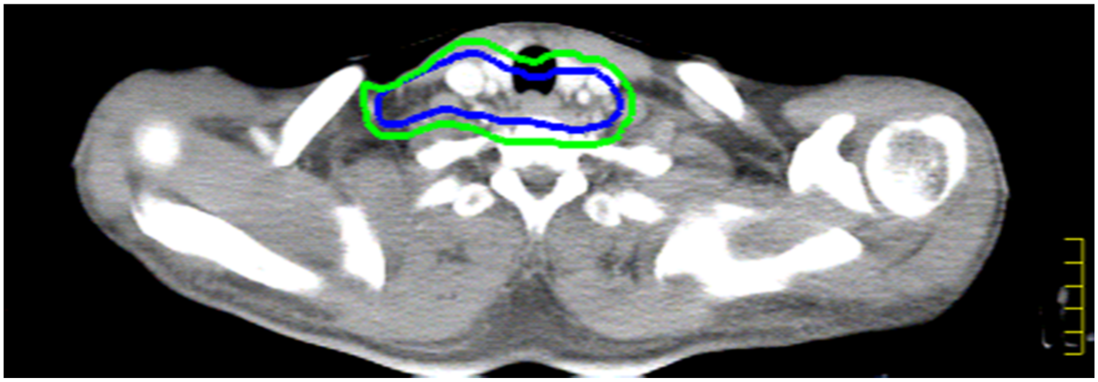

Figure B1. Prevention area in esophageal cancer with supraclavicular lymph node metastasis.

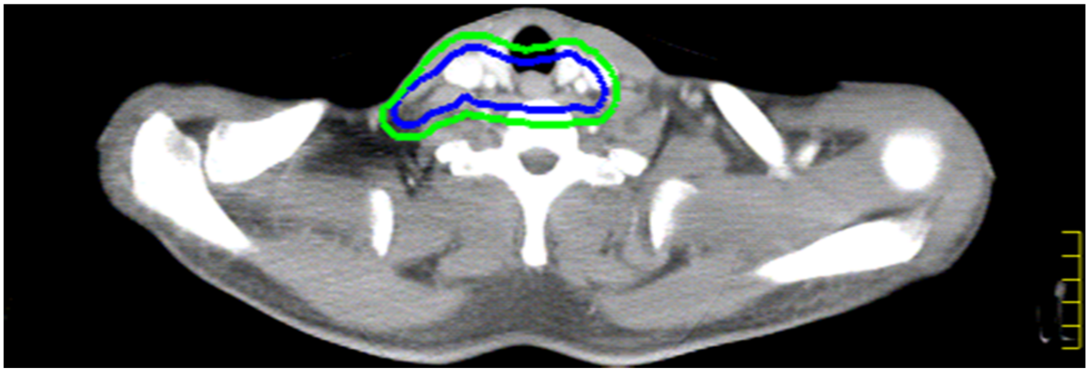

Figure B2. Prevention area in esophageal cancer with supraclavicular lymph node metastasis.

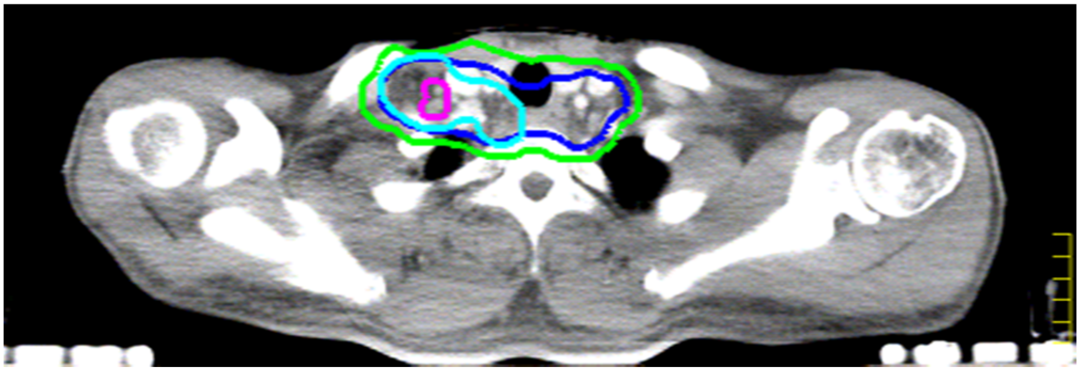

Figure B3. Prevention area in esophageal cancer with supraclavicular lymph node metastasis.

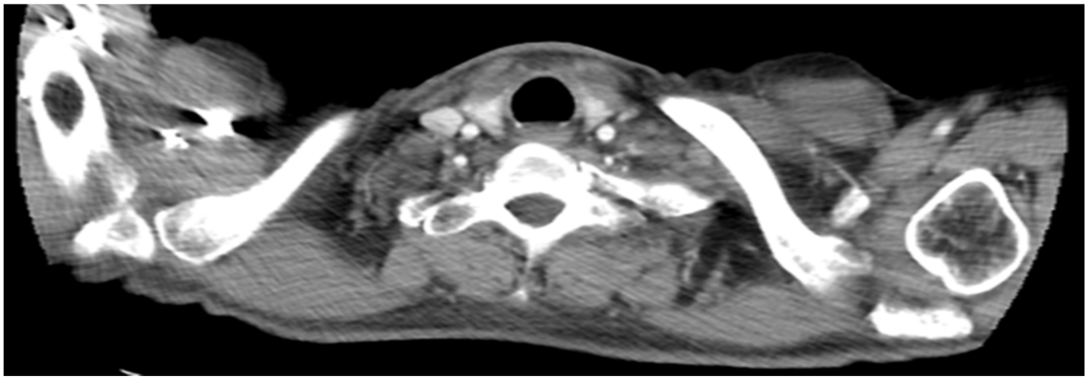

Figure C1. Prevention area in esophageal cancer with supraclavicular lymph node metastasis.

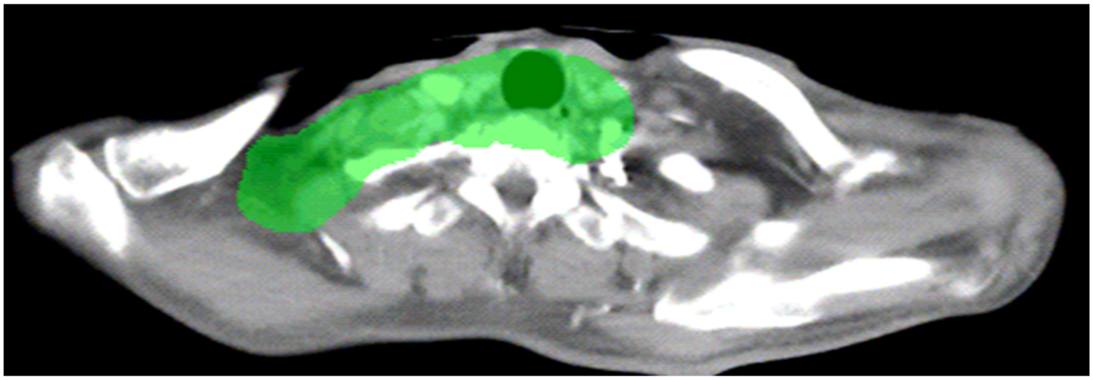

Figure C2. Prevention area in esophageal cancer with supraclavicular lymph node metastasis.

D. Outlining of Normal Tissues and Organs and Evaluation

a) The lungs and heart; the spinal cord, spinal cord PRV, and bone marrow; and the stomach, liver, kidneys, and intestines (the small intestine and colon may be collectively outlined as the bowel) should be outlined for tumors in the lower segment and the esophagogastric junction.

b) The intestines and spinal cord should be outlined to 2 cm above/below the PTV, and the entire organ should be outlined for the lungs, heart, stomach, kidneys, and liver.

c) The spinal cord (*i.e.*, the vertebrae) should be outlined for 1 additional vertebra up and down for the PTV. Only vertebrae should be included, and the transverse processes, spinous process, and intervertebral discs are not included.

E. Dose Limits for Normal Tissues

- *Lungs:* V20 <28%，Dmean <15Gy;
- *Heart:* V30 <40%，V40 <30%;
- *Spinal cord PRV:* Dmax <45Gy;
- *Stomach:* V40 <40%，Dmax <55−60Gy;
- *Small intestines:* V40 <40%，Dmax <55Gy;
- *Kidneys:* V20 <30%;

- *Liver:* V30 <30%.

4) **Concurrent Chemotherapy Regime (Test Group)**

From the day radiotherapy begins, chemotherapy drugs will be given on the day of radiation and administered orally twice a day (BID) at half an hour after a meal, with an interval of 12 hours. No drugs are administered on days with no radiation. The specific oral dose is calculated based on the body surface area:

**Body surface area (m^2)**	**Initial dose per administration**
< 1.25	40 mg
≥1.25 ~ < 1.5	50 mg
≥1.5	60 mg

For example, if a patient's body surface area is 1.8m^2, S-1 is administered orally at 60 mg, BID, on the days of radiation.

5) **Adjuvant Chemotherapy Regime (Test Group):** After radiation therapy is completed, the patient rests for 3–4 weeks, and S-1 is initiated dependent on the hematology and liver and kidney functions (the inclusion criteria of this study must be met). The method of administration and dosage is as follows: The drug is taken for 14 consecutive days and then suspended for 7 days, with 21 days as 1 cycle, for a total of 4 cycles. Hematology will be monitored weekly, and liver and kidney functions tested every 4 weeks during drug administration.

Outline of the Study Design

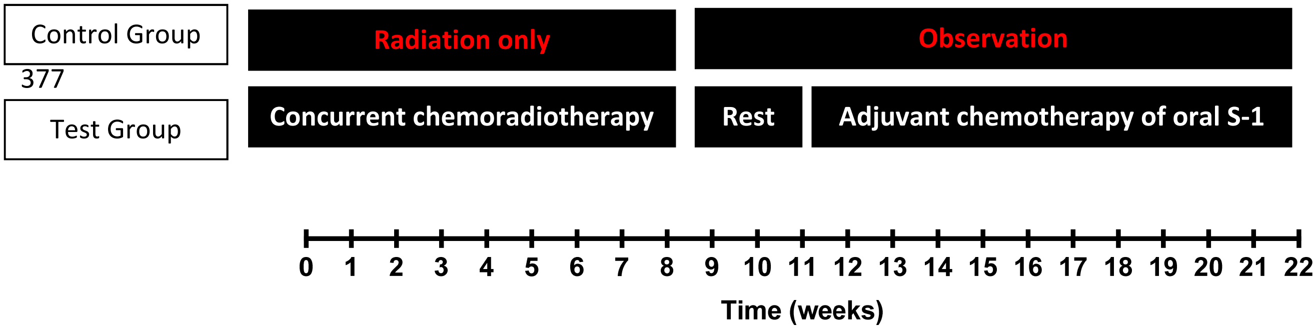

6) **In-Treatment Tests and Assessments**

A. **In-Treatment Tests**

Weekly: General condition (KPS score and body weight records); physical examination; grading of toxic and side reactions; and hematology, re-tested twice a week if abnormal.

B. **Test after Completion of Treatment**

Except for the weekly test items during treatment, the following tests will be performed: a CT scan of the neck, chest, and abdomen (with the same requirements as those for the pre-treatment CT), gastroscopy and intracavitary ultrasounds, CGA, and a comprehensive geriatric assessment and evaluation of the liver and kidney functions.

7) **Management of Adverse Events**

Adverse events during the trial will be graded based on CTCAE 4.0/RTOG, and any adverse events that occur during the trial will be recorded in the CRF. Serious adverse events (SAEs) shall be reported to the Ethics Committee of the hospital in writing within 24 hours, and the patients will be treated promptly. All patients with an SAE will be followed up with until the event is resolved.

According to the principles below, the drug dose will be reduced or even discontinued in case of an adverse event. The treatment details for dose reduction and drug discontinuation will be reflected in the clinical summary. During the trial, if an adverse event occurs, the following principles will be followed, and the details on the specific dose reduction and drug discontinuation of each patient will be recorded in the CRF and summarized in the clinical summary.

Principles for Adverse Events and Dose Reduction/Discontinuation in Chemo- and Radiotherapy

A. **Adverse Events in Chemoradiotherapy**

During this trial, attention will be given to the following conditions during chemoradiotherapy: gastrointestinal reactions, including nausea, vomiting,

abdominal pain, gastritis, dysphagia, reduced appetite, and fatigue; and myelosuppression, mainly reduced platelets.

B. Principles for Dose Reduction/Discontinuation in Chemotherapy

Any Grade 1/2 adverse reactions will be given symptomatic treatment, such as antiemetics, acid suppression, protection of gastric mucosa, and blood-generating medications administered orally or via intramuscular injection.

In case of Grade 4 WBC reduction, Grade 3 adverse reactions in the digestive tract, Grade 2 anemia and platelet reduction, and Grade 2 impaired liver and kidney functions, S-1 will be suspended and symptomatic treatment prescribed. If the adverse event improves to Grade 0−1 within 1 week of treatment, S-1 will be resumed at the original dose. If the adverse event has not improved to Grade 0-1 within this time or if new adverse events of Grade 2 or higher occur, the S-1 treatment and the radiation therapy will be terminated, if necessary. In the entire process, the doses of S-1 and radiation will not be adjusted unless new adverse reactions occur or the original adverse events worsen.

Other Grade 3 adverse reactions will be managed with the same principles for the corresponding Grade 2 adverse reactions.

For any Grade 4 adverse events such as reduction in WBCs or neutrophils, both S-1 and radiotherapy will be discontinued. Radiation may resume when the adverse reaction improves to Grade 0-1, but S-1 will not be resumed.

The principles for drug discontinuation during the adjuvant chemotherapy are the same those described as above.

8) Follow-up and Efficacy Evaluation

A. *Follow-up Assessments:* Follow-up assessments will include the following items:

- Survival status;
- Disease progression of the tumor;
- Local/regional lymph node progression;

- Distant metastasis, including distant lymph node metastasis and hematogenous metastasis;
- Other treatment received (e.g., chemotherapy, surgery, additional radiation therapy);
- Food intake and QoL; and
- Late-onset reactions to radiotherapy.

B. *Efficacy Evaluation:* Short-term (within 1 month after radiotherapy) efficacy of radiation therapy of tumor response evaluation will be measured using CR + PR rate (evaluation criteria RECIST version 1.1 combined with barium meal imaging).

C. *Follow-up Times and Data Collection:* All patients will be followed up every 3 months within 2 years after the radiation therapy is completed, then every half a year, and then once a year after 5 years. The follow-ups shall include the following data collection:

- Medical history, including whether the patients experienced cough, chest tightness, fever, and dysphasia;
- Hematology, chemistry, and tumor marker examinations; and
- Enhanced CTs on the neck, chest, and abdomen; ultrasounds of the neck and abdomen; esophagography and bone ECTs; and, if necessary, brain CTs or MRIs and aspiration cytology.

Information from the follow-ups will be recorded in detail, including late-onset toxicity and recurrence, metastasis, and death.

9) Data Management

A. Data Lock

After the database has been established and confirmed to be correct, the raw data will be locked by the principal investigator, project leads, and statistical personnel. No changes will be made to the data after the lock. Any problems identified after the database is locked will be amended during the statistical analysis once they have been confirmed.

B. Data Processing

After the study data have been entered and the database is locked, the database will be handed over to the statistical personnel for analysis according to the statistical analysis plan. After this analysis is completed, the statistical personnel will write a statistical analysis report. The study personnel, statistical personnel, and sponsor will unblind the study for the second time, and the results will be handed to the principal investigator for the completion of the study report.

C. Data Sets

a) Full Analysis Set

The full analysis set (FAS) is an ideal set of cases that includes the intent-to-treat (ITT) population, and it comprises all randomized patients, with the unreasonable cases excluded. The FAS in this study comprises all patients who have been assigned to treatment groups through randomization, who have received treatment per the protocol of this study, and who have had at least 1 baseline evaluation and 1 post-baseline evaluation.

For patients who have dropped out during the trial, their missing data at the post-dropout observation time points will be managed by carrying forward the corresponding last observation data (last observation carried forward, LOCF); for the missing data for the patients who have completed the trial, the corresponding non-missing data of the nearest time point will be carried forward. For patients who have dropped out after randomization and enrollment with no evaluable post-treatment data, the missing data will not be input and will be processed as is.

b) Per-Protocol Set

The per-protocol set (PPS), also called *effective cases, effective samples,* or *evaluable case samples,* is a data set comprised of the subset of patients with adequate compliance with the protocol such that the data will show the treatment effect according to the scientific model on which the study is based. In this study, the PPS will comprise all randomized

and enrolled patients who adhered to the protocol, who have full baseline values for the primary indicators, who have good compliance, and who complete the trial according to the study protocol. The missing data will not be input and will be processed as is.

c) **Safety Analysis Set**

The safety analysis set comprises all subjects who have been randomized, received at least 1 treatment, and undergone at least 1 safety evaluation.

The compliance of the data sets above involves considerations such as treatment received, measurable primary indicators, and no major violation of the protocol.

10) Statistical Analysis Plan

A. **General Principle:** The statistical analysis will be conducted with the internationally accepted SPSS software, version 20.0. All statistical tests involved in this study will adopt the two-sided significance test, with $p \leq 0.05$ having statistical significance.

B. **Descriptive Statistics and Equilibrium Analysis of the Data**

The basic patient information in both groups, such as gender, age, cancer stage, and tumor length, will be analyzed with descriptive statistics, and the equilibrium of the inter-group distribution will be evaluated. The inter-group comparison of numeric variables, such as age and body weight, will be conducted with the *t* test if the data follow a normal distribution and with the Wilcoxon rank-sum test if otherwise. The inter-group comparison of variables such as gender and stage will be conducted with Pearson's chi-square test, Fisher's exact test, or the CMH test. The analysis will be based on the FAS data. The overall dropout rates and the dropout rates due to adverse events for the two patient groups will be compared with a chi-square test.

For continuous variables, a parametric test (*e.g.,* t test, variance analysis) will be used if the data follow a normal distribution, and a non-parametric test (*e.g.,* rank-sum test) will be used if the data are not normally distributed, If non-normally distributed data become normally distributed after data

transformation, a parametric test method may be used. For categorical variables, a chi-square test may be used for unordered data in a two-way R x C table, with the Fisher's exact test conducted when the number of cells with a theoretical frequency <5 exceeds 1/5 of the total number of cells. For ordered data in a one-way table, a rank-sum test will be used for analysis.

C. Analysis of Efficacy Endpoints

Primary and secondary endpoints are evaluated in the intention-to-treat (ITT) population (all randomized patients). In case of the treatment completion rate is lower than 75%, χ^2 test is used to evaluate whether the missingness was at random or not at random. When the missingness is confirm as missing at random, for missingness imputation, weighted log-rank testing of the ITT population will be conducted to simulate the condition in which the randomization proportion between RT group and CRT-CT group was 1: 1, and survival analysis of the per-protocol (PP) population (randomized participants who completed treatment as planned) will be applied.

D. Analysis of Safety Endpoints

The incidence of adverse events and side effects such as changes in laboratory results will be analyzed. Adverse events will be categorized as adverse reactions and unrelated adverse events. From a conservative perspective for this study, all adverse reactions will be determined to be "definitely related," "probably related," or "possibly related" to the study drug under investigation in the trial. Incidence of adverse events (or adverse reactions) = number of patients with at least 1 adverse event (or adverse reaction)/number of patients with safety.

E. Analysis of Dropout Rates

The overall dropout rates and those due to adverse events for both groups will be analyzed and compared with a chi-square test/Fisher's exact test.

11) Quality Control for the Clinical Trial

A. During this study, a clinical monitor designated by the sponsor will visit the study hospitals regularly for on-site inspection to ensure that all contents of

the study protocol are strictly followed and that the study information is accurately recorded.

- All staff involved in the study will be trained uniformly, and the training method and evaluation criteria will be recorded uniformly. The entire clinical trial will be conducted under the condition of strict blindness.
- The investigators will complete the CRF according to the requirements by filling in each item truthfully, carefully, and in detail to ensure the form's accuracy and reliability.
- Whether a laboratory test result is abnormal is determined by the testing facility's normal reference range.
- All observations and findings in the clinical trial will be verified to ensure the reliability of the data and to ensure that all conclusions of the clinical trial are based on the raw data. Appropriate data management measures will be in place during the clinical trial and when the data are processed.
- Potential dropouts will be actively managed with proper actions.

B. Quality Control and Quality Assurance

- Professional personnel designated by the leading project site will review the enrollment status, entry status of the electronic CRF, and quality of each site. Any problems identified will be promptly communicated.
- Eligibility for the trial will be determined by each site, and the study patients will be informed of the benefits and risks of the treatment and the potential toxic and side effects. The patients must sign the informed consent form to participate. The patients will be assigned to treatments according to the randomization envelopes generated by the physician responsible for randomization at the leading site.
- The chief physician of each site will be responsible for the ward inspection system. During the weekly ward inspection, the chief physician will listen to the medical history, read all information for each patient, modify the target area in person, and carry out the treatment plan according to the assignment.
- A senior residential doctor or attending doctor will position the patients during CT simulation and outline the target areas, which will then be

modified by the chief physician, and the prescribed dose will be administered according to the study requirements.

- A ward inspection system for the radiation therapy department provides the best quality control. Once the research project is established, the project lead will describe the requirements of and details for their department. The weekly ward inspection system will make certain that each newly enrolled patient is inspected during the ward inspection so that the radiation target area, radiotherapy plan, CRF entries, and electronic CRF entries are reviewed.
- Each newly enrolled patient will be verified for radiotherapy before radiation begins, and the physicists will review information, such as the planned radiation dose. All responsibilities will be clearly defined to ensure that the treatment plans are executed without errors.
