# Supplementary material for: Effectiveness of S-1–Based Chemoradiotherapy in Patients 70 Years and Older With Esophageal Squamous Cell Carcinoma: A Randomized Clinical Trial
Source: JAMA Netw Open. 2023 May 17;6(5):e2312625. doi: 10.1001/jamanetworkopen.2023.12625 (PMC10193185; doi:10.1001/jamanetworkopen.2023.12625)
Supplement: Supplement 2. — eTable 1. Causes of Treatment Discontinuity in RT and CRTCT Groups eTable 2. Causes of Death in RT and CRTCT Groups eTable 3. Baseline Characteristics of Patients Enrolled Before February 19, 2019 eTable 4. Baseline Characteristics of Patients Enrolled After February 19, 2019 eTable 5. Incidence of Severe Adverse Events (Grade 3-5) eFigure. Overall Survival and Progression-Free Survival of Patients Who Completed Concurrent Chemotherapy With or Without Consolidated Chemotherapy [file jamanetwopen-e2312625-s002.pdf]

Supplemental Online Content

Wang X, Han W, Zhang W, et al; Jing-Jin-Ji Esophageal and Esophagogastric Cancer Radiotherapy Oncology Group (3JECROG). Effectiveness of S-1–based chemoradiotherapy in patients 70 years and older with esophageal squamous cell carcinoma: a randomized clinical trial. *JAMA Netw Open*. 2023;6(5):e2312625. doi:10.1001/jamanetworkopen.2023.12625

eTable 1. Causes of Treatment Discontinuity in RT and CRTCT Groups
eTable 2. Causes of Death in RT and CRTCT Groups
eTable 3. Baseline Characteristics of Patients Enrolled Before February 19, 2019
eTable 4. Baseline Characteristics of Patients Enrolled After February 19, 2019
eTable 5. Incidence of Severe Adverse Events (Grade 3-5)
eFigure. Overall Survival and Progression-Free Survival of Patients Who Completed Concurrent Chemotherapy With or Without Consolidated Chemotherapy

This supplemental material has been provided by the authors to give readers additional information about their work.

© 2023 Wang X et al. *JAMA Network Open*.

eTable 1. Causes of Treatment Discontinuity in RT and CRTCT Groups

Causes	RT arm	CRTCT arm	*P* value[a]
	No. (%)	No. (%)	
Refuse to complete treatment	13 (39.4%)	52 (53.1%)	0.51
Adverse events	13 (39.4%)	24 (24.5%)	
Comorbidity	5 (15.2%)	17 (17.3%)	
Died or disease progressed before treatment completed	0 (0.0%)	1 (1.0%)	
Outbreak of COVID-19	1 (3.0%)	2 (2.0%)	
Recurrence before consolidated chemotherapy completed	1 (3.0%)	2 (2.0%)	

[a]confirmed by Fisher's exact test.

eTable 2. Causes of Death in RT and CRTCT Groups

Causes	RT arm	CRTCT arm
	No. (%)	No. (%)
Esophageal carcinoma	85 (84.2%)	78 (75.0%)
Comorbidity	9 (8.9%)	17 (16.3%)
Complication	4 (4.0%)	5 (4.8%)
Accidence	2 (2.0%)	2 (1.9%)
Secondary primary tumor	0 (0.0%)	1 (1.0%)
Causes of unknown	1 (1.0%)	1 (1.0%)

© 2023 Wang X et al. *JAMA Network Open.*

eTable 3. Baseline Characteristics of Patients Enrolled Before February 19, 2019

Characteristics		RT (*N*=113)	CRT+CT (*N*=127)	χ2	*P* value
		No. (%)	No. (%)		
Age	＜80 years	86(76.1)	102(80.3)		
	≥80 years	27(23.9)	25(19.7)	0.62	0.43
	Median (IQR)	76.0 (73.0-79.0)	75.0 (72.0-78.0)		
Sex	male	68(60.2)	89(70.1)	2.59	0.11
	female	45(39.8)	38(29.9)		
ECOG performance score	0	5(4.4)	12(9.4)	2.29	0.13
	1	108(95.6)	115(90.6)		
Charlson comorbidity index	0	89(78.8)	88(69.3)		
	1	16(14.2)	29(22.8)	3.18	0.20
	2	8(7.1)	10(7.9)		
Mini nutritional assessment score	＜17.0	16(14.2)	25(19.7)		
	17.0-23.5	62(54.9)	67(52.8)	1.36	0.51
	≥24.0	35(31.0)	35(27.6)		
	Median (IQR)	22.0 (18.8-24.0)	21.5 (17.0-24.0)		
Body mass index	＜18.5	14(12.4)	17(13.4)		
	18.5-23.9	63(55.8)	70(55.1)	0.05	0.97
	≥24.0	36(31.9)	40(31.5)		
	Median (IQR)	22.8 (19.8-24.9)	22.0 (20.3-24.5)		
Pretreatment dietary Intake	general diet	22(19.5)	12(9.4)		
	soft diet	33(29.2)	42(33.1)	7.50	0.06
	fluid diet	56(49.6)	65(51.2)		
	aphagosis	2(1.8)	8(6.3)		
Tobacco consumption	no	68(60.2)	78(61.4)	0.04	0.84
	yes	45(39.8)	49(38.6)		
Alcohol consumption	no	97(85.8)	108(85.0)	0.03	0.86
	yes	16(14.2)	19(15.0)		
Tumor location	upper third	34(30.1)	50(39.4)		
	middle third	55(48.7)	50(39.4)	2.66	0.27
	lower third	24(21.2)	27(21.3)		
Length	＜5.0cm	53(46.9)	41(32.3)		
	≥5.0cm	60(53.1)	86(67.7)	5.36	0.02
	Median (IQR)	5.0 (3.9-6.0)	5.0 (4.0-7.0)		
T stage	T1	4(3.5)	2(1.6)		
	T2	11(9.7)	14(11.0)	-	0.80[a]
	T3	68(60.2)	79(62.2)		
	T4	30(26.5)	32(25.2)		
N stage	N0	30(26.5)	38(29.9)	0.34	0.56
	N1	83(73.5)	89(70.1)		
M stage	M0	99(87.6)	109(85.8)	1.21	0.55

© 2023 Wang X et al. *JAMA Network Open.*

	M1a	6(5.3)	11(8.7)		
	M1b	8(7.1)	7(5.5)		
TNM stage	IIA	20(17.7)	27(21.3)		
	IIB	9(8.0)	11(8.7)		
	III	70(61.9)	71(55.9)	1.98	0.74
	IVA	6(5.3)	11(8.7)		
	IVB	8(7.1)	7(5.5)		
Radiotherapy	incomplete	8(7.1)	14(11.0)	1.12	0.29
	completed	105(92.9)	113(89.0)		
Concurrent chemotherapy	incomplete	-	34(26.8)	-	-
	completed	-	93(73.2)		
Adjuvant chemotherapy	incomplete	-	65(51.2)	-	-
	completed	-	62(48.8)		

[a]confirmed by Fisher's exact test.

© 2023 Wang X et al. *JAMA Network Open*.

eTable 4. Baseline Characteristics of Patients Enrolled After February 19, 2019

Characteristics		RT (*N*=33)	CRT+CT (*N*=57)	χ2	*P* value
		No. (%)	No. (%)		
Age	＜80 years	27(81.8)	43(75.4)	0.49	0.48
	⩾80 years	6(18.2)	14(24.6)		
	Median (IQR)	75.5 (73.0-78.8)	76.0 (72.0-79.0)		
Sex	male	24(72.7)	39(68.4)	0.19	0.67
	female	9(27.3)	18(31.6)		
ECOG performance score	0	4(12.1)	7(12.3)	-	1.00[a]
	1	29(87.9)	50(87.7)		
Charlson comorbidity index	0	27(81.8)	35(61.4)	4.18	0.12
	1	3(9.1)	13(22.8)		
	2	3(9.1)	9(15.8)		
Mini nutritional assessment score	＜17.0	4(12.1)	8(14.0)	0.84	0.66
	17.0-23.5	13(39.4)	27(47.4)		
	⩾24.0	16(48.5)	22(38.6)		
	Median (IQR)	23.3 (20.8-24.5)	22.0 (19.8-25.8)		
Body mass index	＜18.5	5(15.2)	9(15.8)	0.08	0.96
	18.5-23.9	19(57.6)	34(59.6)		
	⩾24.0	9(27.3)	14(24.6)		
	Median (IQR)	22.1 (19.6-23.8)	22.4 (20.0-24.1)		
Pretreatment dietary Intake	general diet	5(15.2)	6(10.5)	-	0.29[a]
	soft diet	16(48.5)	23(40.4)		
	fluid diet	11(33.3)	28(49.1)		
	aphagosis	1(3.0)	0(0.0)		
Tobacco consumption	no	19(57.6)	34(59.6)	0.04	0.85
	yes	14(42.4)	23(40.4)		
Alcohol consumption	no	21(63.6)	50(87.7)	7.28	0.008
	yes	12(36.4)	7(12.3)		
Tumor location	upper third	10(30.3)	21(36.8)	0.95	0.62
	middle third	15(45.5)	20(35.1)		
	lower third	8(24.2)	16(28.1)		
Length	＜5.0cm	11(33.3)	23(40.4)	0.44	0.51
	⩾5.0cm	22(66.7)	34(59.6)		
	Median (IQR)	5.0 (4.0-7.8)	5.0 (4.0-6.0)		
T stage	T1	0(0.0)	0(0.0)	-	0.07[a]
	T2	4(12.1)	2(3.5)		
	T3	19(57.6)	45(78.9)		
	T4	10(30.3)	10(17.5)		
N stage	N0	12(36.4)	23(40.4)	0.14	0.71
	N1	21(63.6)	34(59.6)		
M stage	M0	28(84.8)	54(94.7)	-	0.16[a]

© 2023 Wang X et al. *JAMA Network Open.*

	M1a	3(9.1)	3(5.3)		
	M1b	2(6.1)	0(0.0)		
TNM stage	IIA	7(21.2)	20(35.1)		
	IIB	3(9.1)	1(1.8)		
	III	18(54.5)	33(57.9)	-	0.09[a]
	IVA	3(9.1)	3(5.3)		
	IVB	2(6.1)	0(0.0)		
Radiotherapy	incomplete	5(15.2)	6(10.5)	-	0.52[a]
	completed	28(84.8)	51(89.5)		
Concurrent chemotherapy	incomplete	-	16(28.1)	-	-
	completed	-	41(71.9)		
Adjuvant chemotherapy	incomplete	-	22(38.6)	-	-
	completed	-	35(61.4)		

[a]confirmed by Fisher's exact test.

© 2023 Wang X et al. *JAMA Network Open*.

eTable 5. Incidence of Severe Adverse Events (Grade 3-5)

	RT (*N*=142)	CRT+CT (*N*=176)		
Adverse events	No. (%)	No. (%)	χ2	*P* value
Anemia	1(0.7%)	2(1.1%)	-	1.00[a]
Leukopenia	8(5.6%)	15(8.5%)	0.98	0.32
Neutropenia	5(3.5%)	9(5.1%)	0.47	0.49
Thrombocytopenia	0(0.0%)	2(1.1%)	-	0.50[a]
Nausea	5(3.5%)	15(8.5%)	3.34	0.07
Vomiting	1(0.7%)	6(3.4%)	-	0.14[a]
Fatigue	1(0.7%)	5(2.8%)	-	0.23[a]
Diarrhea	0(0.0%)	1(0.6%)	-	1.00[a]
Esophagitis	8(5.6%)	21(11.9%)	3.76	0.05
Pneumonitis	6(4.2%)	9(5.1%)	0.14	0.71
Fever	0(0.0%)	4(2.3%)	-	0.13[a]
Weight loss	1(0.7%)	1(0.6%)	-	1.00[a]
Transaminitis	0(0.0%)	2(1.1%)	-	0.50[a]

[a]confirmed by Fisher's exact test.

© 2023 Wang X et al. *JAMA Network Open.*

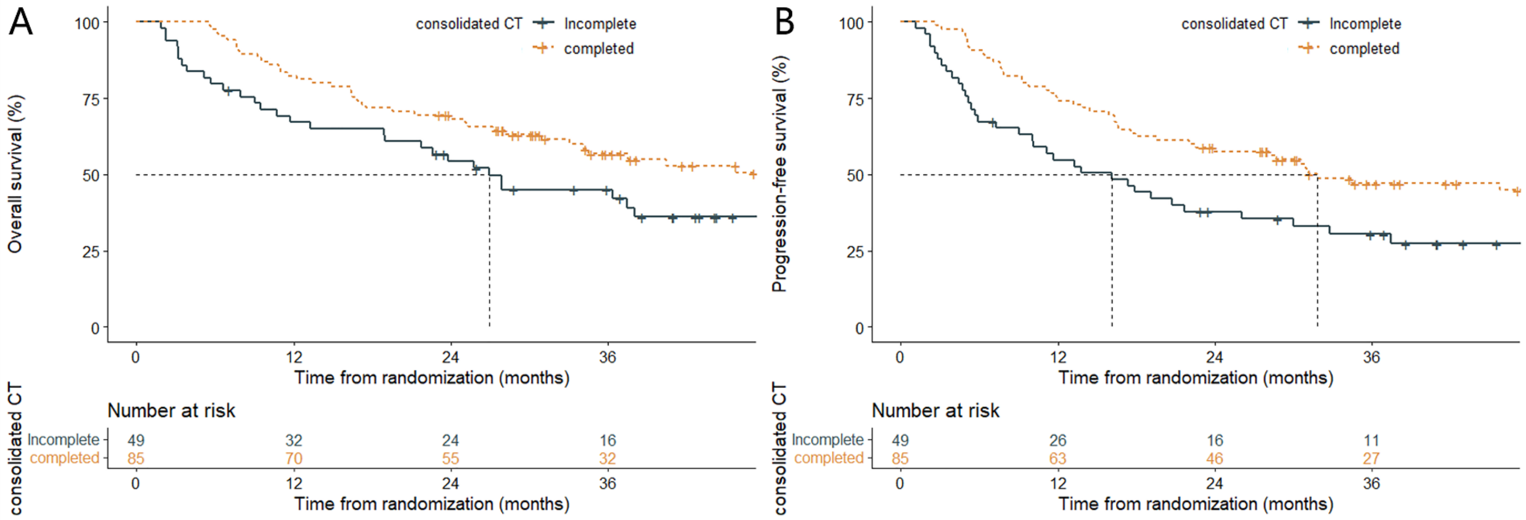

eFigure. Overall Survival and Progression-Free Survival of Patients Who Completed Concurrent Chemotherapy With or Without Consolidated Chemotherapy

A. Overall survival of patients completed concurrent chemotherapy with/ without consolidated chemotherapy (Hazard ratio=0.61, 95% CI: 0.35-0.98, Log-Rank P value=0.04).

B. Progression-free survival of patients completed concurrent chemotherapy with/ without consolidated chemotherapy (Hazard ratio=0.55, 95% CI: 0.35-0.86, Log-Rank P value=0.008).

© 2023 Wang X et al. *JAMA Network Open*.
