# Supplementary material for: Effectiveness of S-1–Based Chemoradiotherapy in Patients 70 Years and Older With Esophageal Squamous Cell Carcinoma: A Randomized Clinical Trial
Source: JAMA Netw Open. 2023 May 17;6(5):e2312625. doi: 10.1001/jamanetworkopen.2023.12625 (PMC10193185; doi:10.1001/jamanetworkopen.2023.12625)
Supplement: Supplement 3. — Members of the Jing-Jin-Ji Esophageal and Esophagogastric Cancer Radiotherapy Oncology Group (3JECROG) [file jamanetwopen-e2312625-s003.pdf]

Supplemental Online Content: Nonauthor Collaborators
*First name, last name, and suffix (if applicable) are required and will appear in PubMed.

*Group Name(s): Jing-Jin-Ji Esophageal and Esophagogastric Cancer Radiotherapy Oncology Group (3JECROG)							
***First Name and Middle Initial(s)**	***Last Name**	***Suffix (eg, Jr, III)**	Academic Degrees	Institution	Location (city, state/province, country)	Role or Contribution, eg, chair, principal investigator	Group (if more than 1 Group listed in the byline) and/or Subgroup (eg, Steering Committee)
Yuanji	Xu		M.D.	Department of Radiation Oncology, Clinical Oncology School of Fujian Medical University, Fujian Cancer Hosptial	Fuzhou, China	Research Coordinator	
Haoying	Wang		M.D.	Department of Radiation Oncology, Anyang Cancer Hospital	Anyang, China	Research Coordinator	
Tian	Zhang		M.D.	Department of Radiation Oncology, Tianjin Medical University Cancer Institute and Hospital/National Clinical Research Center for Cancer	Tianjin, China	Research Coordinator	
Xi	Chen		M.D.	Department of Radiation Oncology, Tianjin Medical University Cancer Institute and Hospital/National Clinical Research Center for Cancer	Tianjin, China	Research Coordinator	
Xiaojie	Xia		M.D.	Department of Radiation Oncology, Nanjing Medical University First Affiliated Hospital	Nanjing, China	Research Coordinator	
Zhigang	Zhang		M.D.	Department of Radiation Oncology, the First Affiliated Hospital of Zhengzhou University	Zhengzhou, China	Research Coordinator	
Fen	Zhang		M.D.	Department of Oncology, Tengzhou Central People’s Hospital	Tengzhou, China	Research Coordinator	
Xiaojing	Sun		M.D.	Department of Radiation Oncology, Affiliated Hospital of Hebei University	Baoding, China	Research Coordinator	

Supplemental Online Content: Nonauthor Collaborators
*First name, last name, and suffix (if applicable) are required and will appear in PubMed.

***First Name and Middle Initial(s)**	***Last Name**	***Suffix (eg, Jr, III)**	Academic Degrees	Institution	Location (city, state/province, country)	Role or Contribution, eg, chair, principal investigator	Group (if more than 1 Group listed in the byline) and/or Subgroup (eg, Steering Committee)
Fei	Teng		M.D.	Department of Radiation Oncology, Affiliated Hospital of Hebei University	Baoding, China	Research Coordinator	
Yu	Lin		M.D.	Department of Radiation Oncology, The Affiliated Hospital of Inner Mongolia Medical University	Hohhot, China	Research Coordinator	
Shufeng	Zhang		M.D.	Department of Radiation Oncology, The Affiliated Hospital of Inner Mongolia Medical University	Hohhot, China	Research Coordinator	
