# Supplementary material for: Effectiveness of S-1–Based Chemoradiotherapy in Patients 70 Years and Older With Esophageal Squamous Cell Carcinoma: A Randomized Clinical Trial
Source: JAMA Netw Open. 2023 May 17;6(5):e2312625. doi: 10.1001/jamanetworkopen.2023.12625 (PMC10193185; doi:10.1001/jamanetworkopen.2023.12625)
Supplement: Supplement 4. — Data Sharing Statement [file jamanetwopen-e2312625-s004.pdf]

Data Sharing Statement

Wang. Effectiveness of S-1–Based Chemoradiotherapy in Patients 70 Years and Older With Esophageal Squamous Cell Carcinoma. *JAMA Netw Open*. Published May 17, 2023. doi:10.1001/jamanetworkopen.2023.12625

Data
Data available: Yes
Data types: Deidentified participant data
How to access data: All pertinent data to support this study and additional, related documents are included in the paper and further data are available via the corresponding author (Zefen Xiao, xiaozefen@sina.com) after approval of a proposal, with a signed data access agreement.
When available: With publication

Supporting Documents
Document types: Statistical/analytic code
How to access documents: All pertinent data to support this study and additional, related documents are included in the paper and further data are available via the corresponding author (Zefen Xiao, xiaozefen@sina.com) after approval of a proposal, with a signed data access agreement.
When available: With publication

Additional Information
Who can access the data: researchers whose proposed use of the data has been approved
Types of analyses: for study purpose
Mechanisms of data availability: after approval of a proposal
